# ODF2L acts as a synthetic lethal partner with WEE1 inhibition in epithelial ovarian cancer models

**DOI:** 10.1172/JCI161544

**Published:** 2023-01-17

**Authors:** Jie Li, Jingyi Lu, Manman Xu, Shiyu Yang, Tiantian Yu, Cuimiao Zheng, Xi Huang, Yuwen Pan, Yangyang Chen, Junming Long, Chunyu Zhang, Hua Huang, Qingyuan Dai, Bo Li, Wei Wang, Shuzhong Yao, Chaoyun Pan

**Affiliations:** 1 Department of Obstetrics and Gynecology, The First Affiliated Hospital,; 2 Department of Biochemistry and Molecular Biology, Zhongshan School of Medicine, and; 3 Advanced Medical Technology Center, The First Affiliated Hospital, Zhongshan School of Medicine, Sun Yat-sen University, Guangzhou, China.

**Keywords:** Oncology, Cancer

## Abstract

WEE1 has emerged as an attractive target in epithelial ovarian cancer (EOC), but how EOC cells may alter their sensitivity to WEE1 inhibition remains unclear. Here, through a cell cycle machinery–related gene RNAi screen, we found that targeting outer dense fiber of sperm tails 2–like (ODF2L) was a synthetic lethal partner with WEE1 kinase inhibition in EOC cells. Knockdown of ODF2L robustly sensitized cells to treatment with the WEE1 inhibitor AZD1775 in EOC cell lines in vitro as well as in xenografts in vivo. Mechanistically, the increased sensitivity to WEE1 inhibition upon ODF2L loss was accompanied by accumulated DNA damage. ODF2L licensed the recruitment of PKMYT1, a functionally redundant kinase of WEE1, to the CDK1–cyclin B complex and thus restricted the activity of CDK1 when WEE1 was inhibited. Clinically, upregulation of ODF2L correlated with CDK1 activity, DNA damage levels, and sensitivity to WEE1 inhibition in patient-derived EOC cells. Moreover, ODF2L levels predicted the response to WEE1 inhibition in an EOC patient–derived xenograft model. Combination treatment with tumor-targeted lipid nanoparticles that packaged ODF2L siRNA and AZD1775 led to the synergistic attenuation of tumor growth in the ID8 ovarian cancer syngeneic mouse model. These data suggest that WEE1 inhibition is a promising precision therapeutic strategy for EOC cells expressing low levels of ODF2L.

## Graphical abstract

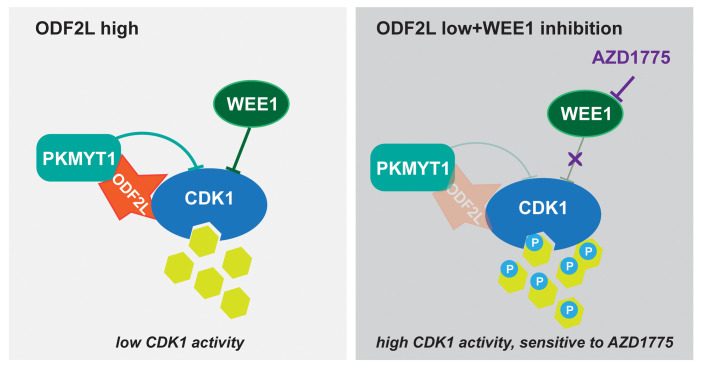

## Introduction

Epithelial ovarian cancer (EOC), the most common subtype of ovarian cancer, ranks fifth in cancer-related deaths among women (1, 2). EOC is more than a single disease, as it has extremely high histological and molecular diversity (3). The first-line treatment in EOC mainly rests on genotoxic platinum-based therapeutics, which are initially effective because of the high levels of replication stress often observed in EOC cells (4). However, platinum resistance eventually occurs in almost all patients with EOC and causes cancer recurrence and death (5, 6). The 5-year survival rate of patients with EOC has not significantly improved for decades (2). Effective strategies, desirably with a broad application spectrum for most patients with EOC, remain an unmet challenge.

Recently, the protein kinase WEE1 has emerged as an attractive target in EOC and multiple other solid tumors (7, 8). The narrow-spectrum inhibitor of WEE1, AZD1775 (also known as adavosertib or MK1775), shows promising antitumor activity as a single agent or in combination with chemotherapies and has been investigated in several phase II clinical trials (9–12). Therapy targeting WEE1 holds great potential for EOC clinical treatment in the near future. WEE1 plays a critical role in maintaining the S- and G_2_/M-phase DNA damage checkpoints by inactivating CDK1 (13, 14). EOC cells are presumably vulnerable to the inhibition of S- and G_2_/M-phase DNA damage checkpoints, owing to the aberrant p53 signaling caused by TP53 mutation, the predominant genetic alteration in human EOC that abrogates the G_1_ checkpoint and renders EOC cells more dependent on S- and G_2_/M-phase DNA damage checkpoints (15). Although some studies have shown that WEE1 inhibition selectively kills or sensitizes p53-deficient cancer cells to chemotherapy, others have found that the antitumor effect of monotherapy or chemosensitization is independent of p53 status (16). Several studies have shown that mTOR activation status significantly affects the sensitivity to WEE1 inhibition in cancer cells (17–19), whereas Pfister et al. reported that H3K36me3-deficient cancers are acutely sensitive to WEE1 inhibition (20). Therefore, insights into the mechanisms that determine the sensitivity to WEE1 inhibition in cancers, especially EOC, are still unclear. Furthermore, clinical studies have revealed that the antitumor benefits of AZD1775 come at the cost of an increased risk of adverse events such as hematologic toxicity (21). Capitalizing on the therapeutic opportunity while maintaining tolerability relies on the targeted use of WEE1 inhibition to minimize toxicity.

In eukaryotes, the protein kinases PKMYT1 and WEE1 are functionally redundant, and both can inhibit the CDK1–cyclin B complex by directly phosphorylating CDK1. WEE1 phosphorylates CDK1 on Y15, whereas PKMYT1 phosphorylates CDK1 on both Thr14 and Tyr15 (22). It would be expected that PKMYT1 could compensate for WEE1’s functional loss and therefore promote resistance to WEE1 inhibition. However, some studies report that knockdown or inhibition of WEE1 alone is sufficient to abolish the S- and G_2_/M-phase DNA damage checkpoints, independent of PKMYT1 (23–25), whereas other studies show that upregulation of PKMYT1 confers resistance to WEE1 loss (22, 26, 27). Therefore, the rescue of S- and G_2_/M-phase DNA damage checkpoints following WEE1 inhibition by PKMYT1 is context dependent. An understanding of how PKMYT1 conditionally coordinates with WEE1 to maintain the S- and G_2_/M-phase DNA damage checkpoints in cell cycle regulation is still limited.

In this study, through a cell cycle machinery–related gene RNAi screen followed by multidisciplinary approaches, we identified outer dense fiber of sperm tails 2–like (ODF2L) as a central driver of resistance to WEE1 inhibition in ovarian cancer. ODF2L licensed the recruitment of PKMYT1 to the CDK1–cyclin B complex and mediated the phosphorylation of CDK1 by PKMYT1 upon WEE1 inhibition. We also present a promising therapeutic strategy that targets ODF2L to significantly increase the sensitivity and overcome the resistance to WEE1 inhibition in ovarian cancer.

## Results

### ODF2L expression correlates with sensitivity to WEE1 inhibition in EOC cells.

**Figure 1:**
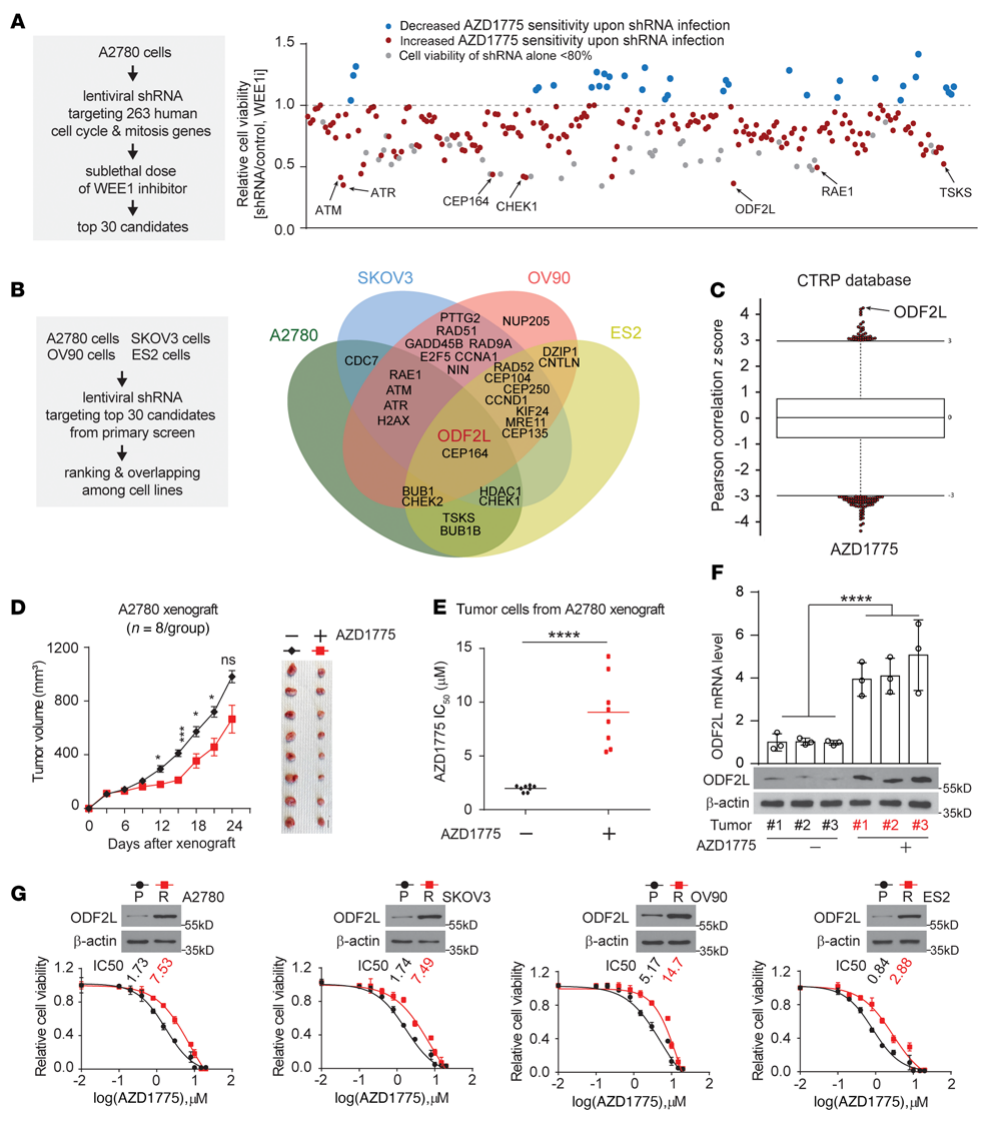
ODF2L expression correlates with sensitivity to WEE1 inhibition in EOC cells. (**A**) Primary screening test of 263 genes in A2780 cells was performed using a sublethal dose (200 nM) of AZD1775 (left). shRNAs that alone induced cell death (>20%, gray) were excluded (right). (**B**) Secondary screen using the top-30 candidates from the primary screen in 4 EOC cell lines (left). Thirty leads based on shRNA and AZD1775 treatment are shown in a Venn diagram (right). (**C**) Plotted data were mined from the CTRP database. The correlation between ODF2L expression and resistance to AZD1775 in ovarian cancer cells is shown. Plotted values are *z* scored Pearson’s correlation coefficients. (**D** and **E**) Development of the in vivo AZD1775-resistant model. A2780 xenograft mice were treated with a high dose of AZD1775 (60 mg/kg; orally, once per day from day 4 to day 10). Tumor volumes were recorded (**D**), and AZD1775 resistance of the xenograft tumors was determined by AZD1775 IC_50_ at the experimental endpoint (**E**). (**F**) RT-qPCR and Western blot results show increased ODF2L expression in A2780 xenograft mouse tumors collected from the AZD1775-treated group (marked in red) compared with the vehicle-treated group. Three representative tumors from each group were randomly selected for analyses. (**G**) AZD1775 IC_50_ in 4 pairs of AZD1775-resistant and parental ovarian cancer cells. The data shown are representative of 2 (**F** and **G**) independent biological experiments. Data are the mean ± SD from 3 technical replicates for **F**. Error bars represent the SEM for tumor volume (**D**). *P < 0.05, ***P < 0.001, and ****P < 0.0001, by 2-way ANOVA (**D**), 1-way ANOVA for (**F**), and unpaired 2-tailed *t* test (**E**).

To gain insight into the mechanisms that affect the sensitivity to WEE1 inhibition in EOC cells, we performed a cell cycle machinery–related gene RNAi screen using a customized lentiviral shRNA library sorted from the TRC lentiviral shRNA library and targeting 263 human cell cycle–related genes represented by 1,295 shRNA constructs (Supplemental Table 1; supplemental material available online with this article; https://doi.org/10.1172/JCI161544DS1). The primary screen involved transducing A2780, a commercially available human ovarian carcinoma cell line, with a lentivirus pool containing shRNAs targeting each of the 263 individual genes and treating them with sublethal doses of AZD1775. From the primary screen using A2780 cells, the top 30 of 263 genes were selected for the secondary validation screen (Figure 1A). The secondary screen evaluated the top-30 candidates in 3 more ovarian cancer cell lines, including SKOV3, OV90, and ES2, in addition to A2780, that encompassed different histologic subtypes and underlying genomic aberrations. shRNAs that alone induced more than 20% cell death were excluded for each specific cell line. ODF2L was identified as the third most effective target from the primary screen and emerged as a lead hit from the secondary screen, as it sensitized cancer cells to WEE1 inhibition across ovarian cancer types (Figure 1B). Moreover, data mined from the Cancer Therapeutics Response Portal (CTRP), a database reporting correlations between gene expression and drug resistance for over 800 cancer cell lines, showed that ODF2L expression most significantly positively correlated with resistance to AZD1775 in ovarian cancer (Figure 1C). In addition, we found that ODF2L protein abundance did not fluctuate during the cell cycle in ovarian cancer cell lines, suggesting that ODF2L expression was possibly not regulated by the cell cycle (Supplemental Figure 1A). To further confirm that ODF2L was involved in the response to WEE1 inhibition in EOC, we established an in vivo AZD1775-resistant model, mimicking the clinical development process of chemotherapy resistance (Figure 1D). Nude mice were inoculated s.c. with A2780 cells. Three days later, when the tumors reached a volume of nearly 100–150 mm^3^, a high dose of AZD1775 (60 mg/kg) or vehicle control was administered orally once per day for 7 days. A2780 xenografts grew slowly in response to AZD1775 for the initial 15 days. However, tumor growth eventually markedly **in**creased in the later stage. Mice were sacrificed for tumor harvesting at the endpoint, when there was no significant difference in tumor volumes between the AZD1775-treated group and the control group (Figure 1D). In contrast, the AZD1775 IC_50_ increased by approximately 4.5-fold in the tumor cells isolated from the AZD1775-treated cells compared with the treatment-naive tumor cells (Figure 1E), indicating the successful induction of resistance to WEE1 induction in vivo. In this in vivo model, we found that the expression of ODF2L was dramatically increased in tumor cells from the AZD1775-treated group, as validated by quantitative reverse transcription PCR (RT-qPCR) and immunoblotting (Figure 1F). Additionally, while the expression level of ODF2L highly correlated with AZD1775 sensitivity in the parental ovarian cancer cells (Supplemental Figure 1B), we also observed a consistent and substantial increase in ODF2L expression in a panel of 4 AZD1775-resistant ovarian cell lines derived from the paired parental cell lines (Figure 1G). In addition, 19 of the 30 genes identified in the RNAi screen functioned sublethally in the specific parental cell lines with WEE1 inhibition (Figure 1B), and their expression specifically increased in the paired resistant cell lines (Supplemental Figure 1C). Taken together, these data demonstrate that ODF2L expression negatively correlated with sensitivity to WEE1 inhibition in ovarian cancer.

### ODF2L acts as a synthetic lethal partner of WEE1 in EOC cells.

**Figure 2:**
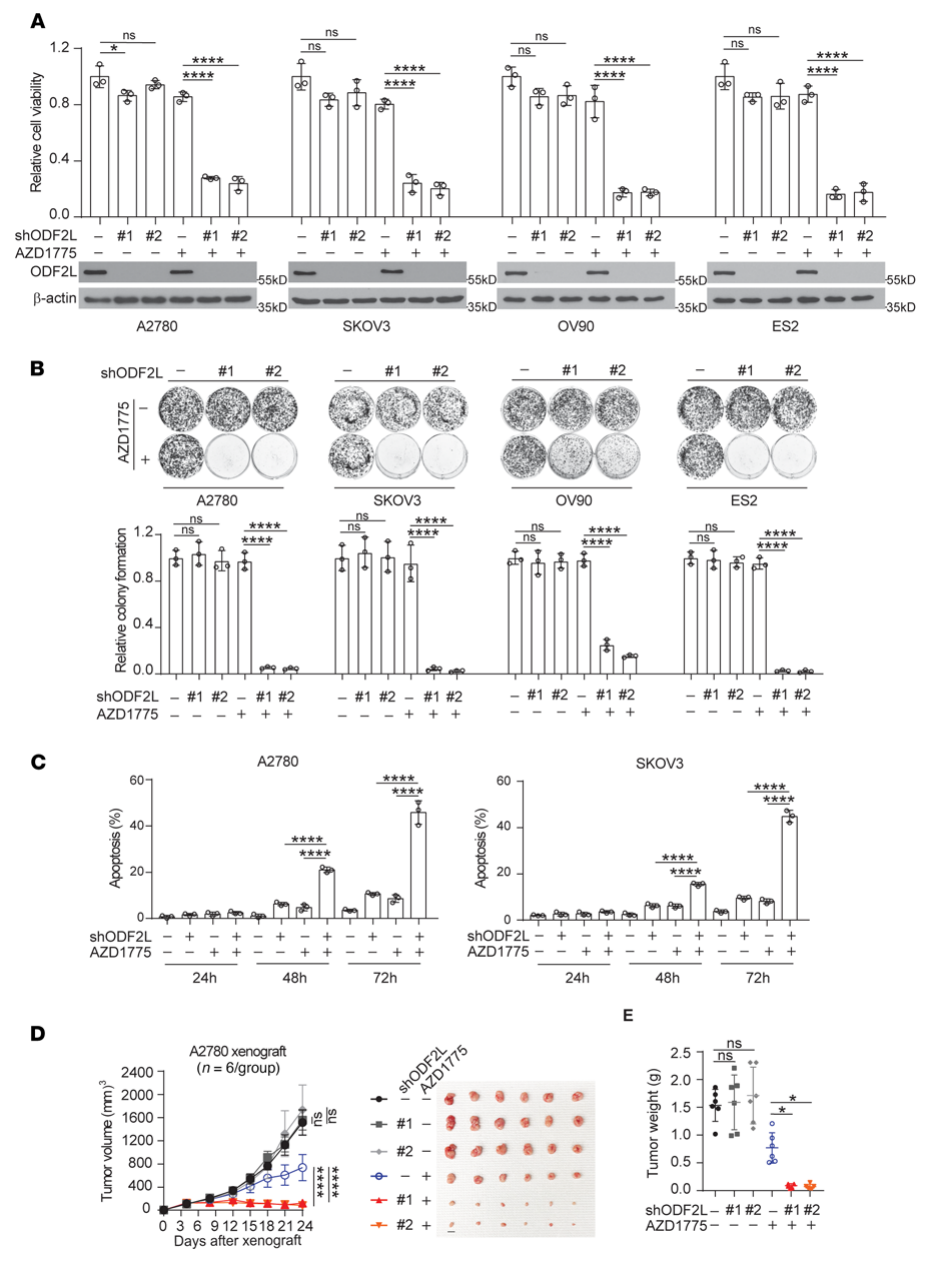
ODF2L acts as a synthetic lethal partner of WEE1 in EOC cells. (**A** and **B**) Cell viability (**A**) and colony formation potential (**B**) of A2780, SKOV3, OV90, and ES2 cells with ODF2L knockdown. Cells were transduced with ODF2L shRNA clones and treated with sublethal doses of AZD1775 (A2780, 200 nM; SKOV3, 200 nM; OV90, 1 μM; ES2, 100 nM) for 72 hours. The knockdown efficiency of ODF2L is shown by immunoblotting. (**C**) Cell apoptosis in A2780 and SKOV3 cells with ODF2L knockdown. Cells were treated with a sublethal dose of AZD1775 for the indicated time points (A2780, 200 nM; SKOV3, 200 nM). (**D** and **E**) Effect of ODF2L knockdown and AZD1775 treatment on tumor growth. Mice were treated with vehicle or AZD1775 (40 mg/kg; orally, once per day) from 3 days after xenografting, and tumor growth (**D**) and tumor weight at the endpoint (**E**) were monitored. Scale bars: 10 mm for tumor size. (**A**–**C**) Data are the mean ± SD from 3 technical replicates of each sample and are representative of 3 (**A**), 2 (**B**), and 3 (**C**) independent biological experiments. (**D** and **E**) Error bars represent the SEM for tumor volume and the SD for tumor weight (*n* = 6). *P < 0.05, **P < 0.01, and ****P < 0.0001, by 2-way ANOVA for tumor volume (**D**) and 1-way ANOVA for all other data.

To investigate the role of ODF2L in the response to WEE1 inhibition in EOC cells, we target-downregulated ODF2L using 2 distinct shRNA clones in 4 EOC cell lines (Figure 2A). Although knockdown of ODF2L alone did not significantly attenuate cell viability or colony-forming potential, ODF2L loss led to significantly decreased cell viability and colony formation following treatment with a sublethal dose of AZD1775) (Figure 2, A and B). In addition, using FACS, we found that ODF2L knockdown significantly aggravated the apoptotic cell death of EOC cells treated with a sublethal dose of AZD1775, further confirming the action of ODF2L as a synthetic lethal partner of WEE1 in EOC cells (Figure 2C and Supplemental Figure 2A). Rescued expression of shRNA-resistant ODF2L restored colony formation and reduced the apoptosis caused by ODF2L knockdown in the presence of AZD1775, excluding the possibility of off-target effects of shODF2L (Supplemental Figure 2, B and C). Moreover, sensitization to AZD1775 treatment upon the loss of ODF2L was also observed in vivo in a xenograft mouse model. ODF2L knockdown caused a significant decrease in tumor growth and tumor size in mice treated with AZD1775 (Figure 2, D and E). These data reveal that ODF2L expression significantly affected the response to WEE1 inhibition in EOC cells.

### ODF2L loss exacerbates DNA damage induced by WEE1 inhibition in EOC cells.

**Figure 3:**
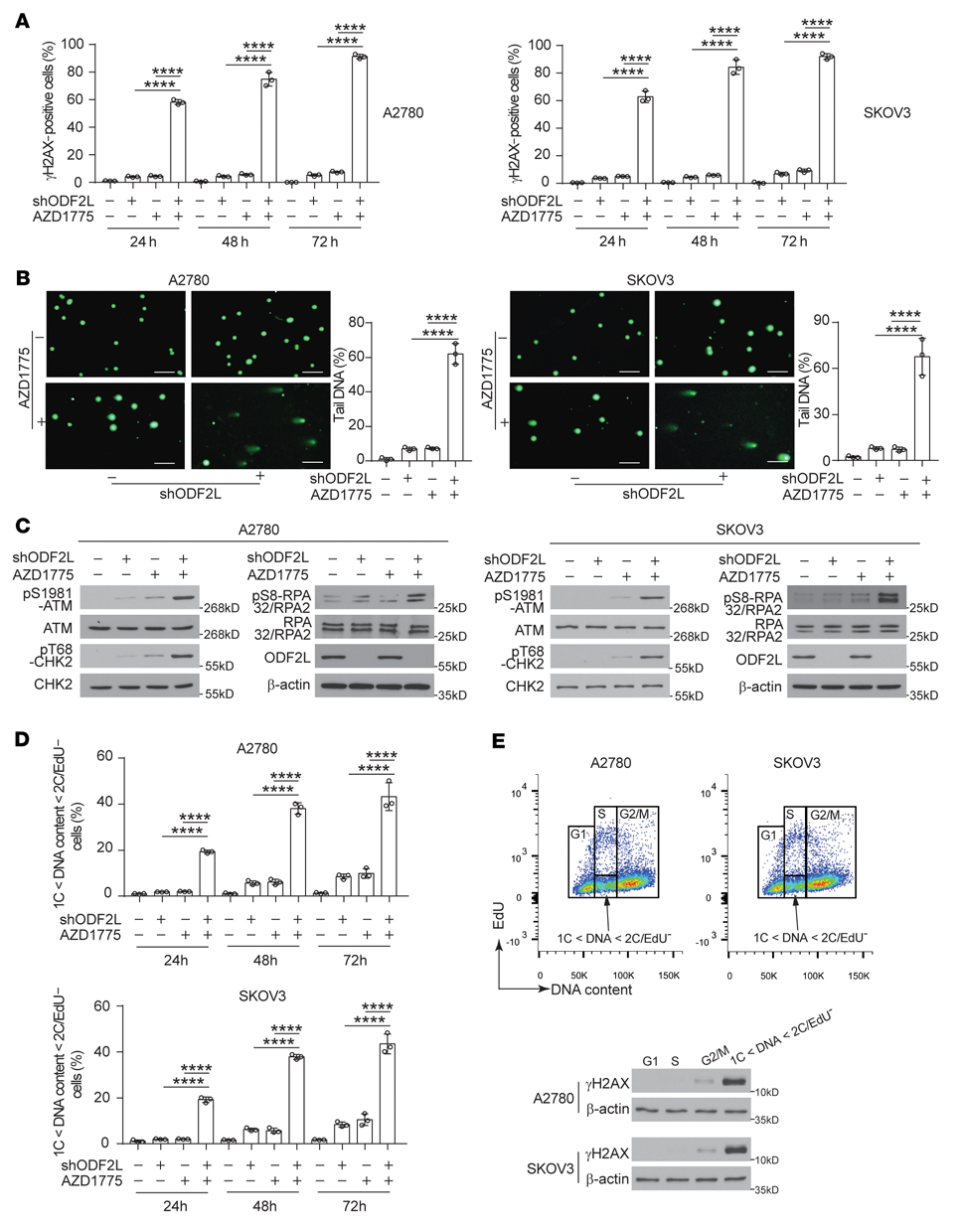
ODF2L loss exacerbates DNA damage induced by WEE1 inhibition in EOC cells. (**A**) Percentages of γH2AX^+^ cells in the indicated EOC cells (treatment: DMSO/200 nM AZD1775) determined by immunofluorescence staining at the indicated time points. (**B**) Representative images of the alkaline comet assay performed in the indicated EOC cells (treatment: DMSO/200 nM AZD1775, 48 hours). For each of the 3 independent experiments, approximately 100 individual cells from 3 random fields were scored for the proportion of DNA in the COMET “tail.” Scale bars: 100 μm. (**C**) Immunoblots of DSB checkpoint proteins in the indicated EOC cells (treatment: DMSO/200 nM AZD1775, 24 hours). (**D**) Flow cytometric analysis of DNA synthesis (EdU) and DNA content (Hoechst 33342) at the indicated time points. The percentages of cells with 1C < PI < 2C/EdU^–^ were quantified. (**E**) Immunoblot analysis of γH2AX levels in cell lysates from the indicated populations of cells (lower panel). The cell populations were sorted by flow cytometry on the basis of cell cycle distribution (upper panel). Data are the mean ± SD from 3 technical replicates of each sample and are representative of 3 (**A**), 2 (**B**), 3 (**C**), 3 (**D**), and 2 (**E**) independent biological experiments. ****P < 0.0001, by 1-way ANOVA for all data.

WEE1 inhibition can promote mitotic entry and increase genomic instability by ectopic activation of CDK1. The premature initiation of mitosis induced by WEE1 inhibition is associated with a progressive accumulation of DNA damage resulting from double-stranded break (DSB) formation, replication fork collapse, and abnormal mitosis. Monoallelic expression of the constitutively active CDK1 T14A/Y15F mutant induced replication stress and cell death in mouse embryonic fibroblasts (MEFs), with a substantial increase in γH2AX levels, chromosomal fragmentation, and DNA damage response (DDR) activation (28). Therefore, to gain insight into the mechanism by which ODF2L sensitized cells to AZD1775 treatment, we first monitored the levels of DNA damage using the DSB marker γH2AX. As shown in Figure 3A and Supplemental Figure 3A, ODF2L knockdown dramatically increased the percentage of γH2AX^+^ cells upon treatment with AZD1775, as measured by immunofluorescence staining in both A2780 and SKOV3 cells. We further validated this observation using an alkaline comet assay and ataxia-telangiectasia mutated kinase–dependent (ATM-dependent) signaling event profiling to monitor DNA damage. Consistent with γH2AX, we observed a significantly increased amount of DNA in the comet tail (Figure 3B) and levels of phosphorylation of ATM, CHK2, and RPA32/RPA2 (Figure 3C) in the ODF2L-knockdown cells relative to control cells upon treatment with AZD1775. In addition, rescued expression of shRNA-resistant ODF2L decreased the percentage of γH2AX^+^ cells, reduced the amount of DNA in the comet tail, and dampened the activation level of ATM-dependent signaling in ODF2L-knockdown cells treated with AZD1775, further confirming the on-target effect of ODF2L in regulating DNA damage. (Supplemental Figure 3, B–D). To test whether the increased DNA damage was a result of combined deficiencies in cell cycle regulation in the ODF2L-knockdown cells with WEE1 inhibition, we monitored the cell cycle distribution using the click chemistry–based EdU assay coupled with the DNA content stain Hoechst 33342. We found that the cell population in G_2_/M phase increased dramatically in ODF2L-knockdown cells treated with AZD1775, implying that the loss of ODF2L coupled with WEE1 inhibition possibly abrogated the G_2_/M checkpoint and forced premature and unscheduled entry into mitosis (Supplemental Figure 4A). Indeed, as shown in Figure 3D and Supplemental Figure 4B, the percentage of the cell population with DNA content between 1C and 2C but EdU^–^ (1C < DNA content < 2C/EdU^–^) was significantly increased in ODF2L-knockdown cells under AZD1775 treatment, which could also be rescued by the forced expression of shRNA-resistant ODF2L in the ODF2L-knockdown cells (Supplemental Figure 4C). These data further support the idea that the combination of ODF2L loss and WEE1 inhibition caused unscheduled entry into mitosis, resulting in a significantly higher number of cells with incompletely synthesized DNA content in mitosis, a deleterious form of DNA damage. Consistent with this result, the cell population with DNA content between 1C and 2C but EdU^–^ (1C < DNA content < 2C/EdU^–^) expressed a substantially higher amount of γH2AX, as determined by immunoblotting following flow cytometric cell sorting (Figure 3E). The above results indicated that ODF2L knockdown promoted dysregulation of the cell cycle and further exacerbated DNA damage induced by WEE1 inhibition in EOC cells.

### ODF2L restrains CDK1 activity induced by AZD1775.

**Figure 4:**
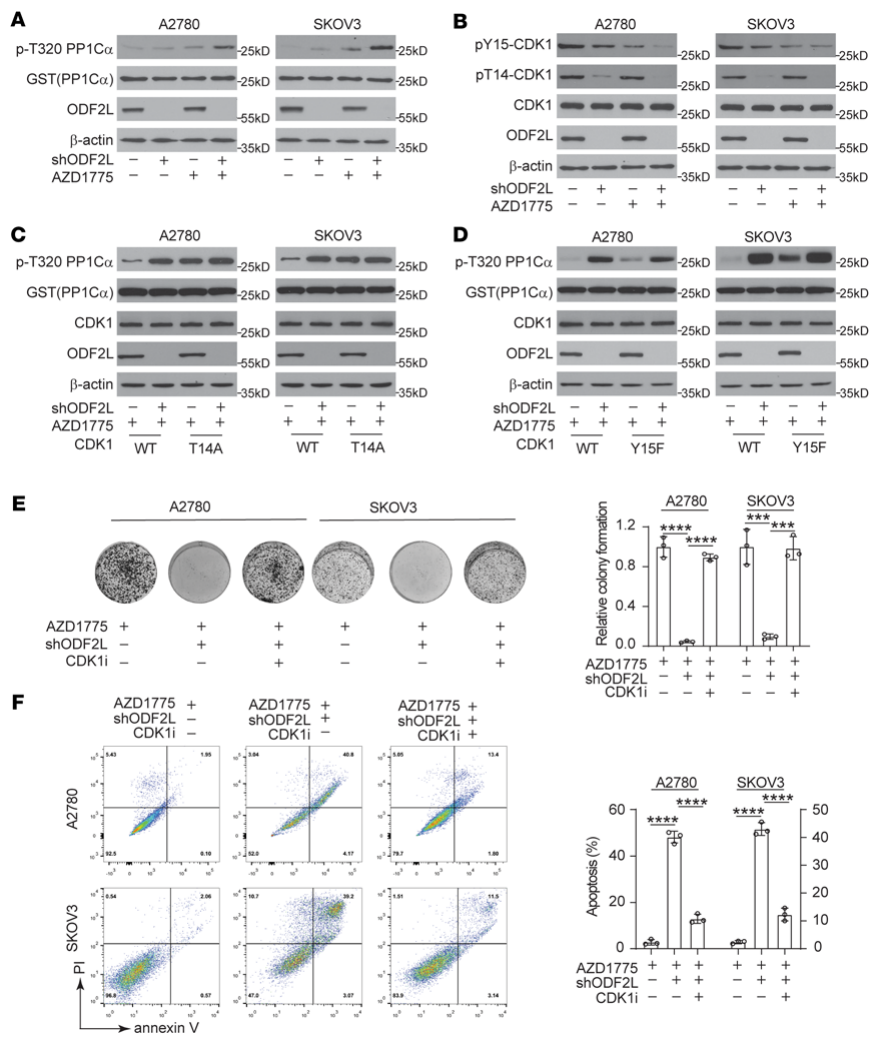
ODF2L restrains CDK1 activity induced by AZD1775. (**A**) In vitro CDK1 activity (top2 plots) was analyzed in EOC cells with or without ODF2L knockdown and then treated with DMSO or AZD1775 (treatment: DMSO/200 nM AZD1775, 4 hours). (**B**) Immunoblots of the T14 and Y15 phosphorylation status of CDK1 in the indicated EOC cells (treatment: DMSO/200 nM AZD1775, 24 hours). (**C** and **D**) In vitro CDK1 activity (top 2 plots) was analyzed in CDK1 T14A–expressing (**C**), CDK1 Y15F–expressing (**D**), and CDK1 WT–expressing (**C** and **D**) EOC cells with endogenous CDK1 removed (treatment: DMSO or 200 nM AZD1775 for 4 hours). (**E** and **F**) Representative images and quantification of colony formation (**E**) and apoptotic cell death (**F**) of EOC cells with or without ODF2L knockdown. Cells were treated with sublethal doses of AZD1775 (A2780, 200 nM; SKOV3, 200 nM) together with or without 5 μM Ro-3306 for 72 hours. Data are the mean ± SD from 3 technical replicates of each sample and are representative of 3 (**A**), 2 (**B**), and 3 (**C**–**F**) independent biological experiments. Blots shown were run in parallel, contemporaneously using the same cell lysate harvested from 1 representative experiment for each cell line in **A**–**D**. ***P < 0.001 and ****P < 0.0001, by 1-way ANOVA.

We next explored the mechanism by which ODF2L promoted dysregulation of the cell cycle and further exacerbated DNA damage induced by WEE1 inhibition in EOC cells. Considering that the main downstream target of WEE1 is the CDK1–cyclin B1 complex, we first checked whether ODF2L reinstated the inactivation of CDK1 when WEE1 was inhibited by monitoring the change in in vitro CDK1 activity. First, ODF2L-knockdown or -overexpressing cells and the indicated control cells were treated with AZD1775 for 4 hours after G_1_–S release. Cell lysates were then extracted and incubated with a recombinant CDK1 substrate, GST-PP1Cα. We then quantified the total levels of pT320 GST-PP1Cα by Western blotting to monitor CDK1 activity. The combination of ODF2L knockdown and WEE1 inhibition enhanced CDK1 activity (Figure 4A) and prolonged the activation status of CDK1 (Supplemental Figure 5A). Phosphorylation of Tyr15 and Thr14 is critical for the control of CDK1 activity, which keeps the CDK1–cyclin B1 complex repressed until the cell approaches mitosis. WEE1 specifically phosphorylates CDK1 on Tyr15, whereas PKMYT1 has dual activity on Tyr15 and Thr14. Interestingly, we observed that ODF2L loss affected the phosphorylation of both Tyr15 and Thr14, with especially strong attenuation of Thr14 phosphorylation in CDK1, strikingly reminiscent of the function of PKMYT1 (Figure 4B). To determine whether ODF2L is important for Thr14 phosphorylation in CDK1, we generated CDK1 T14A–, CDK1 Y15F–, or CDK1 WT–expressing EOC cells with endogenous CDK1 removed and monitored the change in CDK1 activity in these EOC cells under AZD1775 treatment. We found that ODF2L loss markedly increased CDK1 activity in CDK1 WT cells but not in CDK1 T14A cells (Figure 4C). In contrast, increased CDK1 activity was similarly observed in both CDK1 WT and CDK1 Y15F cells when ODF2L was knocked down (Figure 4D), suggesting that ODF2L may specifically signal through CDK1 Thr14 phosphorylation to constrain CDK1 activity under WEE1 inhibition. Indeed, treatment with the CDK1 inhibitor Ro-3306 strongly rescued the colony formation potential (Figure 4E) and decreased apoptotic cell death (Figure 4F) in ODF2L knockdown cells relative to the control cells after WEE1 inhibition. In parallel experiments, we found that cotreatment of ODF2L-knockdown cells with Ro-3306 abolished the AZD1775-induced decline in cell viability in a dose-dependent manner (Supplemental Figure 5B). Taken together, these data suggest that ODFL2 could function to promote CDK1 Thr14 phosphorylation and thus restrain CDK1 activity in the context of WEE1 inhibition in EOC cells.

### ODF2L licenses the recruitment of PKMYT1 to the CDK1 complex.

**Figure 5:**
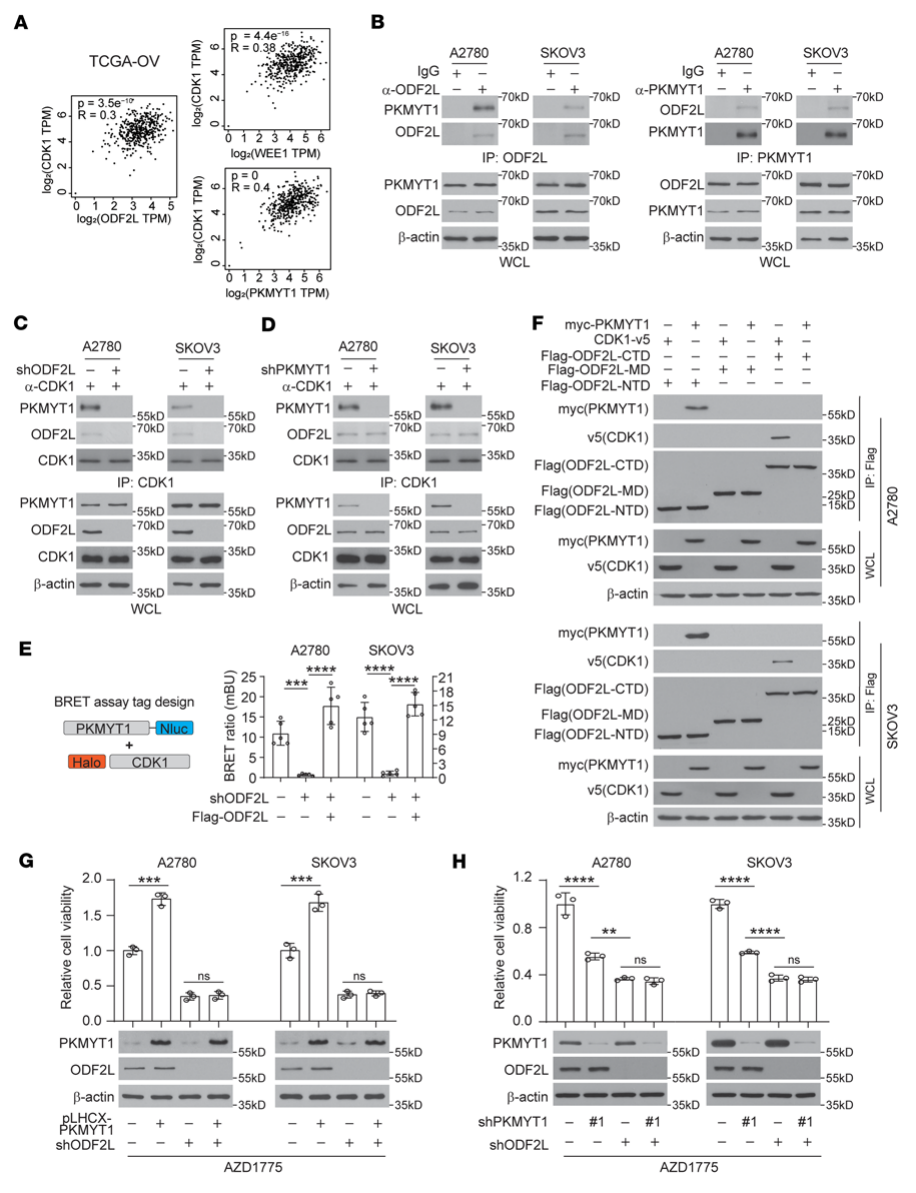
ODF2L licenses the recruitment of PKMYT1 to the CDK1 complex. (**A**) Analysis of correlations between CDK1 expression levels and those of WEE1, PKMYT1, and ODF2L using TCGA OV data. (**B**) Analysis of the endogenous interaction between ODF2L and PKMYT1 by reverse coimmunoprecipitation followed by immunoblotting. (**C** and **D**) Analysis of the interaction between CDK1 and PKMYT1 in ODF2L-knockdown EOC cells (**C**) and the interaction between CDK1 and ODF2L in PKMYT1 knockdown EOC cells (**D**) by immunoprecipitation of endogenous CDK1 followed by immunoblotting. (**E**) BRET assay for the binding of PKMYT1 and CDK1 (plasmid design strategy is shown on left) measured in the indicated cells transfected with Halo-tagged CDK1 and Nluc-tagged PKMYT1 in the absence or presence of ODF2L. (**F**) Analysis of the binding of CDK1 and PKMYT1 to the C-terminally, middle-range, and N-terminally truncated ODF2L by coimmunoprecipitation. The indicated cells were cotransfected with Myc-PKMYT1, CDK1-V5, or Flag-tagged truncated ODF2L domains. (**G** and **H**) Analysis of cell viability upon AZD1775 treatment with or without ODF2L knockdown upon overexpression of PKMYT1 (**G**) or knockdown of PKMYT1 (**H**). AZD1775 (A2780, 200 nM; SKOV3, 200 nM). Data are the mean ± SD from 3 technical replicates in **E**, **G**, and **H**. Data are representative of 2 (**B**), 2 (**C**), 2 (**D**), 3 (**E**), 2 (**F**), 2 (**G**), and 2 (**H**) independent biological experiments. Blots shown were run in parallel, contemporaneously using the same cell lysate harvested from 1 representative experiment for each cell line in **H**. **P < 0.01, ***P < 0.001, and ****P < 0.0001, by 1-way ANOVA.

We next explored how ODF2L promotes the phosphorylation of CDK1 in EOC cells upon WEE1 inhibition. Interestingly, by analyzing The Cancer Genome Atlas (TCGA) ovarian carcinoma (OV) database, we noticed that, similar to the strong correlation between the levels of WEE1 and PKMYT1 and that of their substrate CDK1, the level of ODF2L also significantly correlated with that of CDK1 (Figure 5A). Therefore, ODF2L may participate in the WEE1 or PKMYT1 complex regulating CDK1 phosphorylation. Considering that the role of ODF2L in modulating phosphorylated Thr14 (p-Thr14) of CDK1 overlaps with that of PKMYT1, we first tested whether ODF2L works together with PKMYT1 to regulate CDK1 phosphorylation. The EOC cell lysate was extracted 4 hours after G_1_–S release for immunoprecipitation against ODF2L or PKMYT1. We observed a strong interaction between ODF2L and PKMYT1 (Figure 5B). Moreover, we found that ODF2L knockdown largely diminished the presence of PKMYT1 in the CDK1 immunoprecipitation complex in EOC cells (Figure 5C); in contrast, PKMYT1 knockdown did not significantly affect the level of ODF2L in the CDK1 immunoprecipitation complex in EOC cells (Figure 5D). Consistent with this observation, the in vitro binding of PKMYT1 and CDK1 was strongly attenuated in the absence of ODF2L, suggesting that ODF2L may regulate the phosphorylation of CDK1 by promoting PKMYT1 binding to the CDK1 complex (Supplemental Figure 6A). In addition, we found that the interaction between CDK1 and PKMYT1 in the context of intact cells was also abolished by the knockdown of ODF2L, which could be restored by the rescued expression of ODF2L, as indicated by the bioluminescence resonance energy transfer (BRET) assay in EOC cells cotransfected with Halo-PKMYT1 and Nluc-CDK1 (Figure 5E). Through immunoprecipitation against Flag in EOC cells transfected with plasmids expressing Flag-tagged N-terminal, middle-range, and C-terminal domains of ODF2L, we confirmed that the N-terminal and C-terminal domains of ODF2L directly interacted with PKMYT1 and CDK1, respectively (Figure 5F). Consistently, we found that, while overexpression or downregulation of PKMYT1 in EOC cells increased or decreased the resistance to AZD1775, respectively, knockdown of ODF2L completely abolished the effect (Figure 5, G and H), indicating the critical role of ODF2L in mediating the recovery of the G_2_/M checkpoint by PKMYT1. Interestingly, knockdown of ODF2L alone was more effective at sensitizing AZD1775 than was knockdown of PKMYT1, possibly because residual PKMYT1 could still be recruited and enriched by ODF2L to inactivate CDK1 (Figure 5H). Taken together, our data suggest that ODF2L mediated the interaction of PKMYT1 with the CDK1 complex and thus inactivated CDK1 and G_2_/M checkpoint recovery by PKMYT1 when WEE1 was inhibited in EOC cells.

### ODF2L expression levels are clinically relevant to AZD1775 sensitivity in EOC.

**Figure 6:**
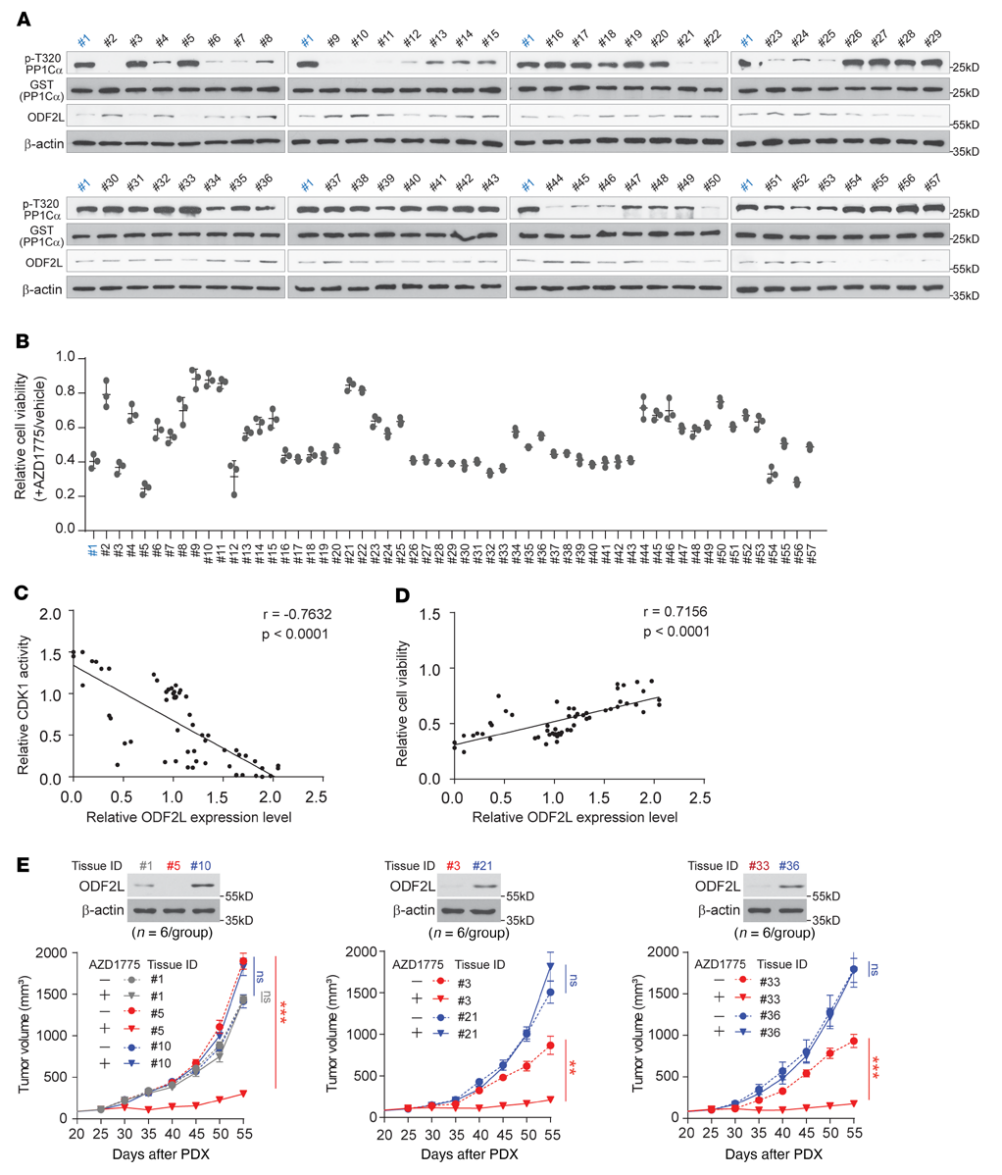
ODF2L expression levels are clinically relevant to AZD1775 sensitivity in EOC. (**A** and **B**) In vitro CDK1 activity, total ODF2L expression levels (**A**) and cell viability (**B**) were analyzed in AZD1775-treated EOC cells derived from the primary tumor tissue of 57 ovarian cancer patients. Patient 1 was used as a control for different batches and labeled blue. (**C** and **D**) Analysis of correlations between ODF2L expression levels and CDK1 activity (**C**) or cell viability (**D**) in AZD1775-treated primary EOC cells. ODF2L expression levels and CDK1 activity were measured by quantification of the blots in **A** using ImageJ software. (**E**) Effect of ODF2L expression levels on in vivo tumor growth of PDXs treated with AZD1775. Tumor tissue from patients 1, 5, and 10; patients 3 and 21; and patients 33 and 36 were chosen for the xenograft on the basis of the differential expression levels of ODF2L in the primary cells confirmed by Western blotting. Mice were evenly grouped when the volume of their tumors reached approximately 100 mm^3^ 25 days after xenografting and were treated with vehicle or AZD1775 (40 mg/kg, orally, once per day). ID, identification. Data are representative of 2 (**A**) and 3 (**B**) independent biological experiments and represent the mean ± SD of 3 technical replicates of each sample (**B**). Error bars in **E** represent the SEM for tumor volume (*n* = 6). **P < 0.01 and ***P < 0.0001, by 2-tailed Pearson’s correlation coefficient (**C** and **D**), 2-way ANOVA for tumor volume (**E**), and 1-way ANOVA for tumor weight (**F**).

To further validate the relationship between ODF2L and the response to WEE1 inhibition in EOC, we examined the correlation of ODF2L levels, CDK1 activity, and cell viability in a panel of AZD1775-treated primary EOC cells derived from 57 tissue samples from patients with ovarian cancer (Supplemental Table 2). To check CDK1 activity, isolated primary cells were treated with AZD1775 for 4 hours following G_1_–S release. Cell lysates were then extracted and incubated with a recombinant CDK1 substrate, GST-PP1Cα. Total levels of p-Thr320 GST-PP1Cα and ODF2L in the total cell lysates were then measured by Western blotting and quantified with ImageJ software (Figure 6A). Meanwhile, we also monitored the viability of the cells treated with AZD1775 for 72 hours (Figure 6B). We found that ODF2L expression levels strongly negatively correlated with CDK1 activity (Figure 6C) and significantly positively correlated with cell viability (Figure 6D) in primary EOC cells treated with AZD1775, indicating the important role of ODF2L in the response of EOC cells to WEE1 inhibition. Additionally, ODF2L expression levels also predicted the varied responses to AZD1775 treatment in vivo in a patient-derived xenograft (PDX) mouse model. Three batches of experiments were performed using primary tumor tissues from patients 1, 5, and 10 (batch 1); patients 3 and 21 (batch 2); and patients 33 and 36 (batch 3), based on the differential expression levels of ODF2L in the primary cancer cells confirmed by Western blotting. AZD1775 treatment resulted in a significant tumor growth delay in the PDX when using the patient cancer tissue with low ODF2L expression, but not the tissue with high ODF2L expression (Figure 6E). Moreover, after normalization by the corresponding vehicle-treated group, tumor growth under AZD1775 treatment was highly correlated with ODF2L expression in the ovarian cancer PDX, indicating a role for ODF2L in affecting the response to WEE1 inhibition in EOC (Supplemental Figure 7, A and B). Taken together, these results strongly support the idea that the ODF2L expression levels are clinically relevant to AZD1775 sensitivity in EOC.

### Targeting ODF2L using an RNAi therapeutic platform sensitizes ovarian cancer cells to WEE1 inhibitor treatment in a syngeneic mouse model.

**Figure 7:**
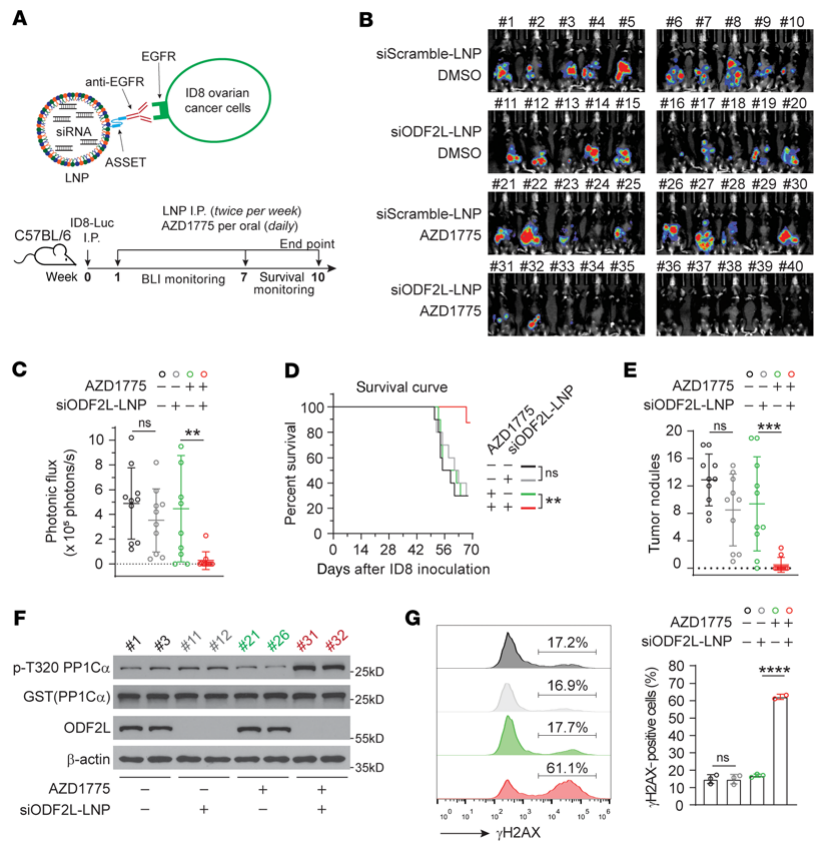
Targeting ODF2L using an RNAi therapeutic platform sensitizes ovarian cancer cells to WEE1 inhibitor treatment in a syngeneic mouse model. (**A**) Schematic illustration of targeted LNP against ovarian ID8 cells using the ASSET platform (upper panel) and experimental design (lower panel). (**B** and **C**) Effect of combination treatment with siODF2L-LNP and AZD1775 on ID8 tumor growth. In vivo bioluminescence image (**B**) and average photonic flux (**C**) at week 7. LNPs (0.75 mg/kg, twice a week); AZD1775 (30 mg/kg, orally, once per day). *n* = 10. (**D**) Survival curves of ID8-bearing mice in the indicated groups. *n* = 10. (**E**) Quantification of peritoneal ID8 tumor nodule numbers in mice at the endpoint. (**F** and **G**) In vitro CDK1 activity and total ODF2L expression levels were analyzed by Western blotting (**F**), and the level of the DNA damage marker γH2AX was analyzed (**G**) by flow cytometry in the 2 or 3 ID8 tumors harvested from mice in each group at the endpoint. Data represent the mean ± SD; *n* = 10 (**B**–**E**). Data are the mean ± SD; *n* = 2 or *n* = 3 (**G**). Data are representative of 2 (**F**) and 3 (**G**) independent biological experiments. **P < 0.01, ***P < 0.001, and ****P < 0.0001, by 1-way ANOVA.

Our finding that ODF2L was a synthetic lethal partner with WEE1 and that loss of ODF2L sensitized the response to WEE1 inhibitors in ovarian cancer indicated that ODF2L could be a promising translational target for ovarian cancer treatment. We evaluated the in vivo therapeutic potential of targeting ODF2L in combination with a WEE1 inhibitor using the ovarian cancer ID8-Luc syngeneic mouse model we previously established (29). To our knowledge, ODF2L is a scaffold protein with no commercially available inhibitor; therefore, we used the targeted RNAi therapeutic lipid nanoparticle (LNP) platform called ASSET (anchored secondary scFv enabling targeting) to specifically knock down ODF2L in ID8 cancer cells in vivo (30, 31). These targeted LNPs are coated with cell-targeting antibodies by binding to a lipid-anchored, single-chain antibody linker that recognizes the Fc region of rat IgG2a (ASSET). Since ovarian ID8 cells specifically and highly expressed the EGFR (32), we coated ODF2L siRNA–loaded (siODF2L-loaded) or scramble siRNA–loaded (siScramble-loaded) LNPs with anti-EGFR antibody and targeted them to ID8 cells (Figure 7A). Mice bearing metastatic peritoneal ID8-Luc tumors were injected i.p. 7 days after tumor inoculation with siScramble-LNPs or siODF2L-LNPs (0.75 mg/kg) conjugated to anti-EGFR (twice per week), together with daily treatment with DMSO or AZD1775 (30 mg/kg). Tumor growth was monitored using in vivo bioluminescence imaging (BLI). The combination of siODF2L-LNPs and AZD1775 significantly abolished tumor growth potential, whereas no significant difference in tumor growth was observed in control mice treated with either vehicle control, siODF2L-LNPs alone, or AZD1775 alone (Figure 7, B and C). Consistently, we observed significantly increased overall survival (Figure 7D), diminished peritoneal hemorrhagic ascites, and reduced tumor nodule numbers (Figure 7E and Supplemental Figure 8A) in the combinatorial group of ODF2L loss and AZD1775 treatment. Treatment with siODF2L-LNPs successfully decreased ODF2L expression in ID8 tumors but not in the main organs, including the heart, liver, spleen, lung, and liver (Figure 7F and Supplemental Figure 8B), resulting in both the substantial activation of CDK1 (Figure 7F) and exacerbation of DNA damage (Figure 7G) in the tumor cells when combined with AZD1775. In addition, the body weight of mice did not fluctuate significantly among any of the groups in the early stage, whereas in the late stage, all mice except those in the combination treatment group lost body weight to a varying extent, possibly due to metastasized cancer (Supplemental Figure 8C). These data provide proof of principle that ODF2L could serve as an effective synthetic lethal target partner with WEE1 inhibitors in the treatment of ovarian cancer.

## Discussion

Here, we identify ODF2L as a synthetic lethal partner of WEE1 that plays a critical role in reinstating AZD1775-resistant G_2_/M cell cycle checkpoint signaling in human EOC cells. Our findings suggest a mechanistic basis by which ODF2L controls EOC cells to evade AZD1775-induced dysregulation of the cell cycle, DNA damage accumulation, and cell death. Cells predominantly depend on CDK1 phosphorylation status for the G_2_/M checkpoint during the cell cycle. Consistently, we found that ODF2L licensed the recruitment of PKMYT1, the functionally redundant kinase of WEE1, to the CDK1–cyclin B complex and mainly phosphorylated CDK1 at Thr14, thereby restricting the activity of CDK1 when WEE1 was inhibited. The reinstatement of CDK1 phosphorylation by PKMYT1-ODF2L reduced DNA damage and cell apoptosis, conferring resistance to WEE1 inhibition. Our studies using clinical samples support that upregulation of ODF2L in primary EOC cells positively correlates with CDK1 inactivation and cell viability in vitro and predicts xenografted tumor growth in vivo under AZD1775 treatment. Furthermore, we demonstrated the in vivo therapeutic potential of targeting ODF2L with siRNA-loaded LNPs combined with AZD1775 using the ovarian cancer ID8-Luc syngeneic mouse model.

Several studies have also reported that WEE1 could be synthetically or selectively targeted to increase cell sensitivity and minimize the toxicity of WEE1 inhibitors. Since WEE1 plays critical roles in maintaining the G_2_/M cell cycle checkpoint, WEE1 inhibition will in theory amplify the effects of DNA damage or related replication stress in the cell cycle, leading to cell death. Indeed, synergistic activity of PARP and WEE1 inhibitors was observed in multiple ovarian cancer models (33). In particular, Fang et al. demonstrated that sequential therapy with PARP and WEE1 inhibitors minimizes toxicity while maintaining efficacy (34). Liang et al. also observed increased sensitivity to AZD1775 in hepatocellular carcinoma and glioma cells with ATRX mutations (35). The combination of ATR and WEE1 inhibitors were also found to have tumor-selective synthetic lethality, leading to tumor remission and inhibited metastasis with minimal side effects in an orthotopic breast cancer model (36). Chen et al. also reported that cyclin E overexpression, which increased replication stress, sensitizes triple-negative breast cancer to WEE1 inhibition (37); a more recent study demonstrated that the amplification of CCNE1, which encodes cyclin E, is synthetically lethal with PKMYT1 kinase inhibition, a functionally redundant kinase of WEE1, emphasizing that the G_2_/M checkpoint is a critical vulnerability in cancers with high replication stress (38). Therefore, in the current study, we performed AZD1775 synthetic lethal RNAi screening targeting 263 human cell cycle–related genes that function to regulate the cell cycle process, mitosis, chromosome segregation, the DDR, and cell cycle checkpoints to identify critical vulnerability across various types of EOC cells and understand how the sensitivity to WEE1 inhibition varies among EOC cells.

The top hit from our screens was ODF2L, a gene without much known function. Our study provides evidence that ODF2L restores the G_2_/M checkpoint by mediating the recruitment of PKMYT1 to the CDK1 complex in EOC cells treated with a WEE1 inhibitor. In theory, WEE1 and PKMYT1 have functionally redundant roles in the inhibition of CDK1 by phosphorylating CDK1 on Tyr15 or on both Thr14 and Tyr15, respectively, thereby controlling the G_2_/M checkpoint together. However, it remains controversial whether PKMYT1 is required for CDK1 inhibition in cells (22). Our study demonstrates that ODF2L expression is a prerequisite for PKMYT1 to restore the G_2_/M checkpoint upon WEE1 inhibition. ODF2L directly interacted with both PKMYT1 and CDK1; ODF2L loss diminished the role of PKMYT1 in the regulation of CDK1 and rescued the G_2_/M checkpoint, sensitizing EOC cells to WEE1 inhibition. Our data also suggest that ODF2L expression increased dramatically in EOCs acquiring resistance to WEE1 inhibition in in vivo and in vitro models, reflecting the dynamic regulation of ODF2L in the recovery of the G_2_/M checkpoint in EOC cells. Future studies are warranted to illustrate the mechanisms that modulate ODF2L expression in EOC cells in response to WEE1 inhibition.

Clinically, our findings indicate that ODF2L could serve as a predictive marker and as a promising therapeutic target to treat patients with EOC in combination with WEE1 inhibitors. We developed an ODF2L-targeted therapy via siRNA-loaded LNPs via a modular platform for targeted RNAi therapeutics, ASSET, and demonstrated proof of therapeutic principle for ODF2L as an effective synthetic lethal target partner with a WEE1 inhibitor in the treatment of EOCs. Combination treatment of ODF2L-targeted therapy via an siRNA-loaded LNP would be expected to benefit patients experiencing severe side effects such as histopathological toxicity after receiving a relatively high dose of a WEE1 inhibitor alone, or patients who have received WEE1 inhibitor–based therapy but whose cancer recurred, in part because of the induction of ODF2L during the treatment. Future studies designed to target ODF2L via chemicals, proteolysis targeting chimeric (PROTAC) degraders, and clinical trials are warranted to fully evaluate and utilize the translational potential of anti-ODF2L therapy in EOC treatment.

## Methods

### Cell culturing.

A2780 and primary ovarian cancer cells were cultured in RPMI 1640 medium with 10% FBS. OV90 cells were cultured in a 1:1 mixture of MCDB 105 medium containing a final concentration of 1.5 g/L sodium bicarbonate and Medium 199 (Gibco, Thermo Fisher Scientific) containing a final concentration of 2.2 g/L sodium bicarbonate with 15% FBS. SKOV3 cells were cultured in McCoy’s 5a medium with 10% FBS. ES2 and 293T cells were cultured in DMEM with 10% FBS. SKOV3, OV90, and ES2 cells were purchased from the American Type Culture Collection (ATCC). A2780 was obtained from MilliporeSigma. All cell lines were authenticated by short tandem repeat (STR) profiling and tested for mycoplasma contamination. AZD1775-resistant sublines of A2780, SKOV3, OV90, and ES2 were generated by serial passage of cells in the presence of increasing concentrations of AZD1775 and maintained in the presence of 50 nM AZD1775. Lentivirus production and stable gene overexpression in human cells were described previously (39, 40).

### Antibodies and the small-molecule inhibitor.

ODF2L (catalog 23887-1-AP) was purchased from Proteintech. PKMYT1 (catalog A302-424A, Thermo Fisher Scientific, for immunoprecipitation) and PKMYT1 (catalog H00009088-B01P, Abnova, for immunoblotting) antibodies were used for immunoprecipitation followed by immunoblotting. In the immunoblots using the whole-cell lysate, antibodies against p-ATM (Ser1981) (catalog 5883); ATM (catalog 2873); p-CHK2 (Thr68) (catalog 2197), CHK2 (catalog 6334); p-RPA32/RPA2 S8 (catalog 83745); RPA32/RPA2 (catalog 35869); p-CDK1 (Tyr15) (catalog 4539); p-CDK1 T14 (catalog 2543); CDK1 (catalog 77055); p–histone γH2AX (Ser139) (catalog 9718); and PKMYT1 (catalog 4282) were all obtained from Cell Signaling Technology. Alexa Fluor 594–conjugated anti–p-histone γH2AX S139 (Ser139) antibody was purchased from BioLegend (catalog 613410). Alexa Fluor 488–conjugated anti–p-histone γH2AX (Ser139) antibody was obtained from MilliporeSigma (catalog 05-636-AF488). The p-PP1Cα T320 (catalog ab62334) antibody was purchased from Abcam. Antibodies against FLAG (catalog F1804/M2, F7425); glutathione S-transferase (GST) (catalog G1160/GST-2); Myc tag (catalog 05-419); and β-actin (catalog A1978/AC-15) were obtained from MilliporeSigma. AZD1775 (Selleckchem; catalog S1525); Ro-3306 (MilliporeSigma; catalog SML0569); and thymidine (MilliporeSigma; catalog T1895) were prepared as 10 mmol/L solutions in DMSO.

### Synthetic lethal RNAi screens.

Primary screening was performed using a lentiviral shRNA library sorted from the TRC Lentiviral shRNA Library obtained from Open Biosystems. A detailed gene list and target sequences are provided in Supplemental Table 1. Cells were seeded into 9 replicates and infected with lentivirus in 96-well plates. Forty-eight hours after infection, cells were treated with DMSO, a sublethal dose of AZD1775 (A2780, 200 nM; SKOV3, 200 nM; OV90, 1μM; ES2, 100 nM), or puromycin (0.5 μg/mL) in triplicate. Cell viability was determined after 3 days of AZD1775 treatment using a CellTiter-Glo Luminescent Viability Assay (Promega). The 30 top-ranking candidates from the primary screen were further confirmed in 3 cell lines — SKOV3, OV90, and ES2 — as performed in the primary screen. Gene candidates that induced more than 20% cell death by shRNA alone and/or had poor shRNA virus transduction efficacy assessed by puromycin selection in each specific cell line were excluded from the final analysis.

### Stable knockdown cell generation.

Stable cells were generated through shRNA virus transduction followed by puromycin selection. Briefly, 2.2 × 10^5^ 293T cells were seeded onto each well of a 6-well plate. After 24 hours, when the cells reached 70% confluence, a mixture of 30 μL solution I containing plasmids (500 ng pLKO.1 shRNA, 500 ng psPAX2, 50 ng pMD2.G dissolved in plain DMEM) and 30 μL solution II (4 μL TransIT-LT1 dissolved in plain DMEM) were incubated for 25 minutes at room temperature and then dropped onto 293T cells for transfection. Beginning 18 hours after transfection, cell media were collected and replaced with fresh complete media twice every 24 hours: the collected media samples were pooled together and filtered through a 0.45 μM polyethersulfone (PES) filter to remove 293T cell debris and harvest the virus. The virus was aliquoted and stored in a –80°C freezer. For infection, cancer cells at 20%–30% confluence in each well of a 6-well plate were incubated with media consisting of 0.3 mL aliquoted virus, 2 μL polybrene (10 mg/mL), and 2.2 mL fresh complete media for 24 hours and then maintained in fresh complete media for another 24 hours. Forty-eight hours after infection, cells were incubated with media containing an optimal concentration of puromycin for selection (A2780: 1.5 μg/mL puromycin, SKOV3: 2.5 μg/mL puromycin). The cells usually reached stability 48–72 hours after selection and were confirmed by Western blotting. ODF2L #1 shRNA target sequence: CCAGTGAAAGTCATCTCAGCT; ODF2L #2 shRNA target sequence: GCGGAGTTGGTAACTCATTCT; ODF2L #1 shRNA was primarily used throughout the study. The following are the #1 shRNA-resistant ODF2L ORF silent mutation primer sequences: forward, CCACGGTGTACCAGTGAGAGCCACCTCAGCTGCCTGAAG; reverse, CTTCAGGCAGCTGAGGTGGCTCTCACTGGTACACCGTGG. The PKMYT1 #1 shRNA target sequence was as follows: CTATGCGGTAAAGCGTTCCAT.

### Cell viability assay and colony formation assay.

Approximately 4,000 cells/well were seeded in 96-well plates 1 day before and then treated with the indicated concentrations of drugs for 72 hours. Cell viability was determined using a CellTiter-Glo Luminescent Viability Assay (Promega) according to the manufacturer’s instructions. For the colony-forming assay, 250 cells were seeded in a 35 mm dish 1 day before and then treated with DMSO or AZD1775 for 72 hours. The treated cells were cultured in fresh complete medium for another 10 days for colony formation. The colonies were stained with 0.5% crystal violet and counted using ImageJ software (NIH).

### Cell synchronization and G_1_/S release.

Cells were synchronized in G_1_/S phase by double thymidine. Rapidly growing cells were treated with 2 mM thymidine for 18 hours twice with a 9-hour release interval between thymidine treatments. After the second round of thymidine treatment, all cells arrested at the G_1_/S boundary and were ready for release into the cell cycle upon incubation with fresh medium.

### CDK1 kinase activity assay.

CDK1 kinase assays were performed as described by Lewis and colleagues (22). Briefly, 20 ng of a 9 amino acid PP1Cα peptide (GRPITPPRN) tagged with GST was combined with cell lysate extract from 2,000 cells in 2× CDK1 phospho-buffer (100 mmol/L β- glycerophosphate, 20 mmol/L MgCl_2_, 20 mmol/L NaF, and 2 mmol/L DTT) with 400 μmol/L ATP and then incubated at 37°C for 15 minutes. Reactions were then terminated with Laemmli sample buffer (Bio-Rad; 1610747) and analyzed by Western blotting.

### Alkaline comet assay.

A Comet Assay Kit (catalog 4250-050-K; Trevigen) was used to perform the alkaline comet assay according to the manufacturer’s instructions. Briefly, a 5 μL volume of cells at 1 × 10^5^/mL was added to 50 μL molten LMAgarose (at 37°C), and 50 μL of mixture was immediately pipetted onto a comet slide. After the mixture of agarose and cells was evenly dispersed, the slides were placed flat at 4°C in the dark for 30 minutes in a high humidity environment. The cells were then lysed overnight by immersing slides in lysis buffer. After lysis, the excess buffer was drained from the slides, and the slides were immersed in freshly prepared alkaline unwinding solution, pH >13, for 1 hour at 4°C in the dark before application of an electric field. The electrophoresis was performed under ice. An electric field (typically 1 V/cm) was applied to the cells for 45 minutes at 4°C, and the cells were stained with SYBR gold (catalog S11494; Thermo Fisher Scientific) for 30 minutes in the dark and photographed using a Zeiss microscope with an attached camera. The comets were analyzed using ImageJ.

### Immunofluorescence.

One milliliter of cells (1 × 10^5^/mL) was seeded onto a microscope coverglass in 12-well cell culture plates overnight and then treated with the indicated concentration of AZD1775 or DMSO for 48 hours. After treatment, the cells were fixed with 4% paraformaldehyde, permeabilized with 0.5% Triton X-100, blocked with normal goat serum, and incubated with Alexa Fluor 488–conjugated anti–p–histone H2A.X (Ser139) antibody (05-636-AF488; MilliporeSigma) for 1 hour at room temperature. Nuclei were counterstained with DAPI (1 mg/mL). Images were obtained using a Zeiss confocal microscope.

### EdU incorporation assay.

A BeyoClick EdU-488 kit (catalog C0071) was used according to the manufacturer’s instructions. Briefly, cells were incubated in a medium with a final concentration of 10 μM EdU for 2 hours at 37°C and then harvested for fixation in 4% paraformaldehyde (PFA) for 15 minutes, followed by washing in PBS and incubation with 0.3% Triton X-100 for 10 minutes, all at room temperature. The cells were then labeled with Azide 488 click addition solution at room temperature for 30 minutes in the dark, rinsed again with PBS, and incubated with 1X Hoechst 33342 for 30 minutes at room temperature. Cell samples were washed with PBS and analyzed or sorted using a BD LSRFortessa flow cytometer.

### Western blotting.

For the fresh cell culture or tissues, total cellular protein was extracted using lysis buffer (50 mM Tris-HCl (pH 7.5), 150 mM NaCl, 1% NP-40, and 0.5% Na-deoxycholate supplemented with Protease Inhibitor Cocktail (Roche). For the PFA-fixed cells sorted using the flow cytometer, a Qproteome FFPE Tissue Kit (catalog 37623; QIAGEN) was used to extract the total protein. Ten percent SDS-PAGE was performed to resolve a total of 30 μg protein, which was transblotted onto a nitrocellulose membrane (Bio-Rad). Membranes were probed with the indicated primary antibodies at 4°C overnight, followed by HRP-conjugated secondary antibodies for 1 hour at room temperature. ECL was used to detect the specific blot bands. Blot bands were quantified by ImageJ software and defined as the ratio of target protein relative to β-actin.

### γH2AX detection by immunofluorescence and flow cytometry.

For immunofluorescence, cells were seeded onto chamber slides (catalog 177380; Nunc Lab-Tek) overnight and treated as indicated. Subsequently, the cells were fixed with 4% PFA for 10 minutes, permeabilized with 0.5% Triton X-100 for 5 minutes, blocked with normal goat serum for 30 minutes, and incubated with Alexa Fluor 488–conjugated anti–p–histone H2A.X (Ser139) antibody (05-636-AF488; MilliporeSigma) for 1 hour at room temperature. Nuclei were counterstained for 10 minutes with DAPI (1 mg/mL; MilliporeSigma). Images were obtained using a laser scanning confocal microscope (LSM 710; Zeiss). For flow cytometry, tumor nodules from 2 or 3 mice were harvested at the endpoint of the ID8 mouse model, diced into approximately 2 mm sections, and crushed with the barrel of a syringe to form homogenate. The homogenate was digested with RPMI 1640 medium containing 1% penicillin-streptomycin, 1× GlutaMAX (catalog 35050061; Thermo Fisher Scientific), and 1% HEPES and type II collagenase (2.5 mg/mL, catalog 17101015; Thermo Fisher Scientific) in a 37°C water bath for 25 minutes and then filtered through a 70 μm filter and spun at 325*g* to pellet the cells. Cells were resuspended and fixed in 4% formaldehyde for 15 minutes at room temperature followed by permeabilization for 10 minutes on ice using ice-cold 100% methanol. Cells were blocked with 0.5% BSA PBS buffer for 30 minutes and incubated with Alexa Fluor 594–conjugated anti–p–histone H2A.X (Ser139) antibody (catalog 613410; BioLegend) for 1 hour at room temperature. Cell samples were analyzed using a BD LSRFortessa flow cytometer.

### RNA extraction and RT-qPCR.

Total RNA was extracted using TRIzol reagent (Invitrogen, Thermo Fisher Scientific), followed by quality and quantity measurements using a NanoDrop ONE (Thermo Fisher Scientific). Total RNA (1 mg) was reverse transcribed to cDNA using the Reverse Transcription Master Kit (Takara) according to the manufacturer’s instructions. RT-qPCR analysis was performed with SYBR green (SYBR Green Supermix; Bio-Rad) in a Bio-Rad CFX Real-Time PCR System. The *ACTB* gene was used as an internal control. The samples were run in technical triplicates.

### Plasmid construction for protein overexpression.

The Gateway cloning strategy was used for overexpression of tagged or nontagged proteins. The specific ORFs of the indicated genes were flag, Myc tagged or nontagged by PCR amplification using Q5 high-fidelity DNA polymerase, and cloned into pENTR/D-TOPO, which was subcloned into pLX302 (C-terminal V5 tag), and a pLHCX-derived Gateway destination vector for overexpression in mammalian cells, or into pET-53-DEST (N-terminal His tag) and pET-60-DEST (N-terminal GST tag) for protein overexpression/purification in bacteria using LR Clonase II (Thermo Fisher Scientific). The primers and clone destination vector for each overexpression plasmid construction are listed in Supplemental Table 3.

### BRET assay.

PKMYT1 and CDK1 were cloned into vectors containing Nluc and Halo fusion tag, respectively, using the Flexi Vector Cloning System (Promega). The indicated cells were transfected with the combination of BRET plasmids for 48 hours and then harvested to measure the bioluminescence according to the manufacturer’s instructions (catalog N1821; Promega).

### In vivo studies.

For all animal studies, animals were randomly chosen, and no statistical method was used to predetermine the sample size. Concealed allocation and blinding of outcome assessment were used.

For the in vivo induction model of AZD-resistant ovarian cancer, nude mice (athymic nu/nu, female, 6 weeks old; Charles River Laboratories) were injected with 0.5 × 10^6^ A2780 cells. Three days after xenograft inoculation, when tumor sizes reached 100 mm^3^, mice were treated with high doses of AZD1775 for only 1 week (60 mg/kg; orally, once a day from day 4 to day 10). Tumor volumes were calculated as 4π/3 × (width/2)^2^ × (length/2).

For the in vivo model to confirm the effect of the loss of ODF2L in sensitizing cells to AZD1775 treatment, nude mice (athymic nu/nu, female, 6 weeks old; Charles River Laboratories) were s.c. injected with 1 × 10^6^ A2780 cells with ODF2L knockdown. Mice were treated with vehicle or AZD1775 (40 mg/kg; orally, once per day) from 3 days after xenograft inoculation when tumor sizes reached 100 mm^3^. Tumor volumes were calculated as 4π/3 × (width/2)^2^ × (length/2).

For the PDX study, tumors from patients with ovarian cancer were implanted into NOD SCIDγ mice (female, 6 weeks old; The Jackson Laboratory). Tumors of approximately 1,500 mm^3^ in size were excised, evenly diced, and implanted into the flanks of nude mice. The mice were randomly divided into groups when the tumor size reached 150 mm^3^, and then AZD175 treatment (40 mg/kg; orally, once per day) was initiated.

For the syngeneic ID8 mouse model, the luciferase gene was transduced into murine ovarian ID8 cells and injected into C57BL/6 mice (female, 6 weeks old; Charles River Laboratories) through i.p. injection. Mice bearing metastatic peritoneal ID8-Luc tumors were injected i.p. 7 days after tumor inoculation with siScramble-LNPs or siODF2L-LNPs (0.75 mg/kg) conjugated to anti-EGFR (twice per week), together with daily treatment with DMSO or AZD1775 (30 mg/kg, orally, once per day). Tumor growth was followed up using in vivo BLI. Fluorescence analysis was conducted using Living Image software (PerkinElmer). Whole blood was harvested at the experimental endpoint for flow cytometric analysis.

Preparation of LNPs and targeted ASSET LNP assembly LNPs were prepared according to a previously described method (31). Briefly, 1 volume of lipid mixture (MC3, DSPC, cholesterol, DMG-PEG, and DSPE-PEG at a 50:10.5:38:1.4:0.1 mol ratio) in ethanol and 3 volumes of siRNA (1:16 w/w siRNA to lipid) in an acetate buffer were injected into a Nanoassemblr microfluidic mixing device (Precision Nanosystems) at a combined flow rate of 2 mL/min (0.5 mL/min for ethanol and 1.5 mL/min for aqueous buffer). The formed LNPs were dialyzed twice against PBS (pH 7.4) for 16 hours to remove ethanol. MC3 (DLin-MC3-DMA) was purchased from Cayman Chemical (catalog 34364). Cholesterol, DSPC (1,2-distearoyl-sn-glycero-3-phosphocholine), DMG-PEG (1,2-dimyristoyl-rac-glycerol-polyethylene glycol), and DSPE-PEG (1,2-distearoyl-sn-glycero-3-phosphoethanolamine-polyethylene glycol) were purchased from Avanti Polar Lipids.

ASSET was produced and purified according to a detailed protocol described previously (30, 31). To incorporate ASSET into LNPs, ASSET was incubated with LNPs for 48 hours at 4°C (1:36, ASSET/siRNA weight ratio). Anti–mouse EGFR antibody (clone ICR10, catalog NB600-724; Novus Biologicals) or a rat IgG2a isotype control (clone 2A3, catalog BE0089; Bio X Cell) was used. The ODF2L siRNA target sequence was as follows: GGGAAGCCGAGAACGATAAGT.

### Statistics.

GraphPad Prism 8 (GraphPad Software) was used to perform the statistical analysis. Data represent the mean ± SD, except for the xenograft tumor growth curves and tumor growth BLI data, which represent the mean ± SEM. No data were excluded. Statistical analysis of significance was based on a 2-tailed Student’s *t* test or 1-way or 2-way ANOVA with Bonferroni’s post hoc multiple-comparison test. *P* values of 0.05 or less were considered statistically significant. Statistical analyses were based on a set of assumptions, such as homogeneity of variances and normal distribution. Variance was similar between the groups that were being statistically compared. No statistical method was used to predetermine sample size; a significant difference was detected in the preliminary studies in our assay, so we used the minimum sample size for all in vivo experiments. In vitro experiments were performed at least 3 times each, per standard practice. Blinding was not performed in this study.

### Study approval.

Animal studies were performed according to protocols reviewed and approved by the IACUC of Zhongshan School of Medicine, Sun Yat-sen University. For the PDX study, informed consent was obtained from each patient, and the experiment was approved by the IRB of Sun Yat-sen University.

## Author contributions

J Li and MX collected clinical tumor samples and performed the histopathological study. WW and S Yao provided clinical information. J Li performed the animal experiments. J Li, J Lu, S Yao, TY, C Zheng, XH, YP, YC, J Long, C Zhang, HH, QD, and BL performed all other experiments. CP designed the study and wrote the manuscript.

## Supplementary Material

Supplemental data

Supplemental table 1

Supplemental table 2

Supplemental table 3

## Version 1 11/15/2022

In-Press Preview

## Version 2 01/17/2023

Electronic publication
