# Supplementary material for: ODF2L acts as a synthetic lethal partner with WEE1 inhibition in epithelial ovarian cancer models
Source: J Clin Invest. 2023 Jan 17;133(2):e161544. doi: 10.1172/JCI161544 (PMC9843051; doi:10.1172/JCI161544)

Supplemental Figures

Supplemental Figure 1. Related to Figure 1. ODF2L expression correlated with sensitivity to WEE1 inhibition in EOC cells. (**A**) Western blot and RT-qPCR analysis of ODF2L expression during cell cycle progression in A2780 and SKOV3 cell lines synchronized by double thymidine following by releasing back into the cell cycle. The cell-cycle profiles were monitored by flow cytometry. (**B**) ODF2L expression and the correlation with AZD1775 IC50 in EOC cells. (**C**) RT-qPCR analysis of the expression level change of all the top 30genes in the resistant cell lines relative to the paired sensitive cell lines. Up-regulated genes were sorted for the Venn diagram. Genes in box indicted synthetic lethality with WEE1 inhibition shown in the figure 2. Data are mean ± SD from 3 technical replicates for A. Statistical analysis was performed by 1-way ANOVA for A and unpaired two-tailed t-test for B (ns, not significant).

Supplemental Figure 2. Related to Figure 2. ODF2L acted as a synthetic lethal partner of WEE1 in EOC cells. (**A**) Representative apoptosis profiles of ODF2L knockdown cells treated with sublethal doses of AZD1775 (A2780, 200nM; SKOV3, 200nM). (**B** and **C**) The effect of ODF2L rescued expression on the colony formation potential (B) and apoptosis (C) of EOC cells with ODF2L knockdown. Cells were treated with sublethal doses of AZD1775 (A2780, 200nM; SKOV3, 200nM; OV90, 1μM; ES2, 100nM) for 72 hours. Rescued level of ODF2L expression is shown by immunoblotting (B, left). Data are mean ± SD from 3 technical replicates for B and C. Statistical analysis was performed by 1-way ANOVA for B and C. (****P < 0.0001)

Supplemental Figure 3. Related to Figure 3. ODF2L loss exacerbated DNA damage induced by WEE1 inhibition in EOC cells. (**A**) Representative image of immunofluorescence staining of γH2AX (green) and DAPI (blue) in the indicated EOC cells (treatment: DMSO/200nM AZD1775, 48hours). Scale bar, 50 μm. (**B**, **C** and **D**) The effect of ODF2L rescued expression on the level of γH2AX (B, Scale bar, 50 μm), DNA in the COMET "tail" (C, Scale bar, 100 μm), and the activation of ATM-dependent signaling (D) in the EOC cells with ODF2L knockdown and AZD1775 treatment. Data are mean ± SD from 3 technical replicates for B and C and are representative of 3 (A), 2 (B), 2(C) and 2(D) independent biological experiments. Statistical analysis was performed by 1-way ANOVA for B and C. (****P < 0.0001)

Supplemental Figure 4. Related to Figure 3. ODF2L loss exacerbated DNA damage induced by WEE1 inhibition in EOC cells. (**A**) Cell cycle distribution in the ODF2L knockdown EOC cells treated with AZD1775 at indicated time points (treatment: DMSO/200nM AZD1775). (**B**) Representative image of flow cytometric profiles of DNA synthesis (EdU) and DNA content (Hoechst 33342) at indicated time points. (**C**) The effect of ODF2L rescued expression on the flow cytometric profiles of

DNA synthesis (EdU) and DNA content (Hoechst 33342) in the EOC cells with ODF2L knockdown and AZD1775 treatment (treatment: DMSO/200nM AZD1775, 72hours). Data are mean ± SD from 3 technical replicates for B and C and are representative of 2 (A), 3 (B), and 3(C) independent biological experiments. Statistical analysis was performed by 1-way ANOVA for C. (****P < 0.0001)

Supplemental Figure 5. Related to Figure 4. ODF2L restrained CDK1 activity induced by AZD1775. (**A**) *In vitro* CDK1 activity was analyzed in EOC cells with or without ODF2L knocked down and then treated with DMSO or AZD1775 at indicated time points (treatment: DMSO/200nM AZD1775). (**B**) Cell viability of vehicle control or ODF2L knockdown cells treated with AZD1775 (200 nM) as a function of CDK1 inhibitor RO3306 dose. Data are mean ± SD from 3 technical replicates for B and are representative of 2 (A), 2(B) independent biological experiments. Statistical analysis was performed by 2-way ANOVA for B. (****P < 0.0001)

Supplemental Figure 6. Related to Figure 5. ODF2L licensed the recruitment of PKMYT1 to the CDK1 complex. (**A**) *In vitro* binding of CDK1 and PKMYT1 in the presence or absence of ODF2L. Left panel, Coomassie blue staining and immunoblot confirmation of indicated protein purified from 293T cells transfected with overexpressing plasmids; Right panel, *in vitro* incubation of the indicated protein and immunoprecipitation of GST-CDK1 followed by glutathione elution and immunoblot. Data are representative of 2 (A) independent biological experiments.

Supplemental Figure 7. Related to Figure 6 ODF2L expression level was clinically relevant to AZD1775 sensitivity in EOC. (**A-B**) Correlation of the ODF2L expression and tumor growth under the WEE1 inhibition in the ovarian cancer PDX models. Tumor volume in the figure 6E were normalized to the corresponding non-AZD1775-treated group (A), and the Pearson's correlation coefficient of between tumor volume and ODF2L expression was analyzed (B).

Supplemental Figure 8. Related to Figure 7 Targeting ODF2L using RNAi therapeutics platform sensitized ovarian cancer cells to WEE1 inhibitor treatment in a syngeneic mouse model. (**A**) Representative images of peritoneal hemorrhagic ascites and tumor nodules in mice at endpoint of each group. (**B**) Relative level of ODF2L mRNA level in the main organs collected in mice at endpoint. The β-actin was used as internal control, n=10. (**C**) Comparation of body weight change in mice, n=10. Statistical analysis was performed by 1-way ANOVA for B and 2-way ANOVA for C (ns, not significant; ****P < 0.0001)

Supplemental Figure1

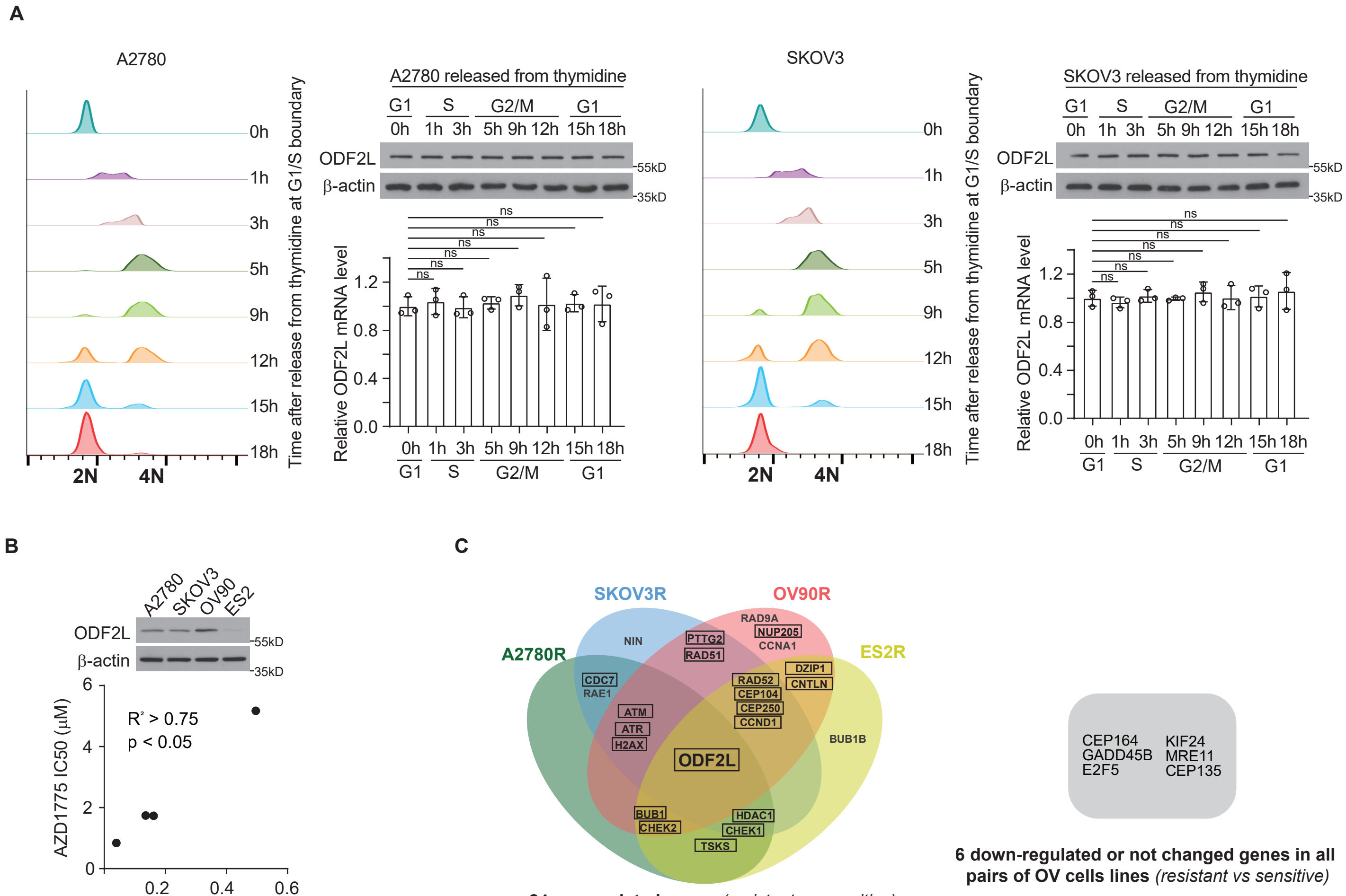

24 up-regulated genes *(resistant vs sensitive)*
(box gene indicated synthetic lethality with AZD1775)

6 down-regulated or not changed genes in all pairs of OV cells lines *(resistant vs sensitive)*

Supplemental Figure 2

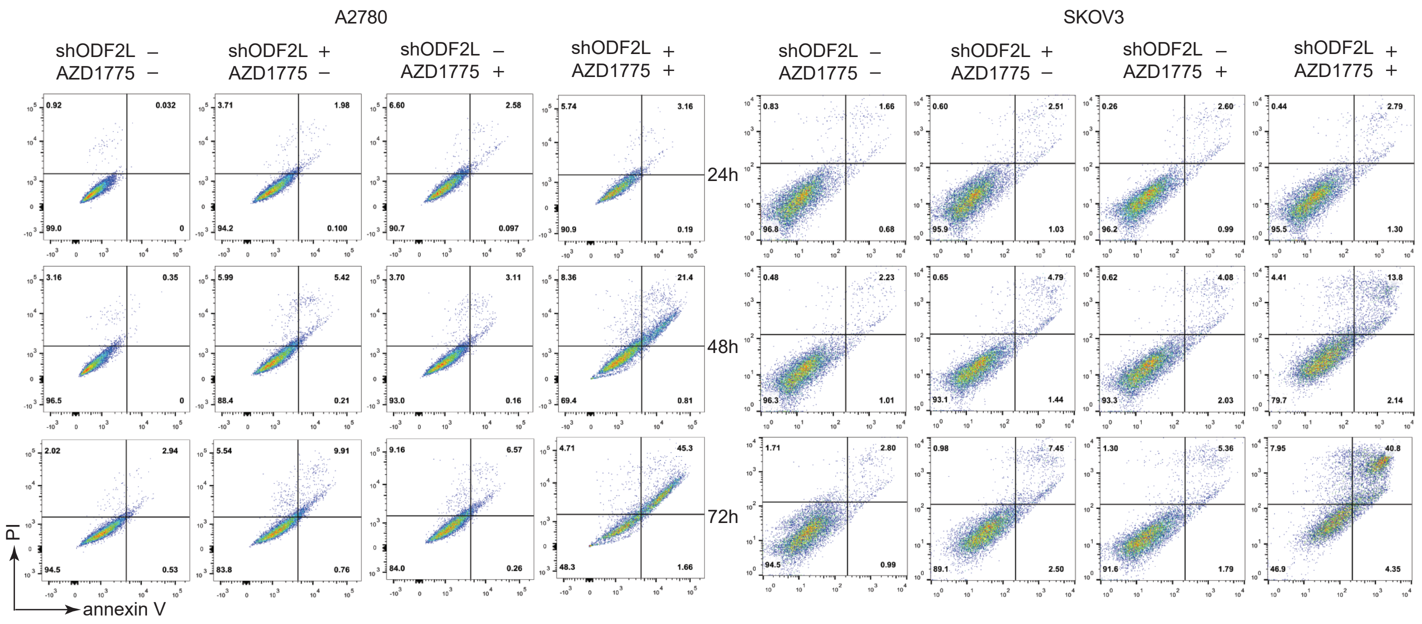

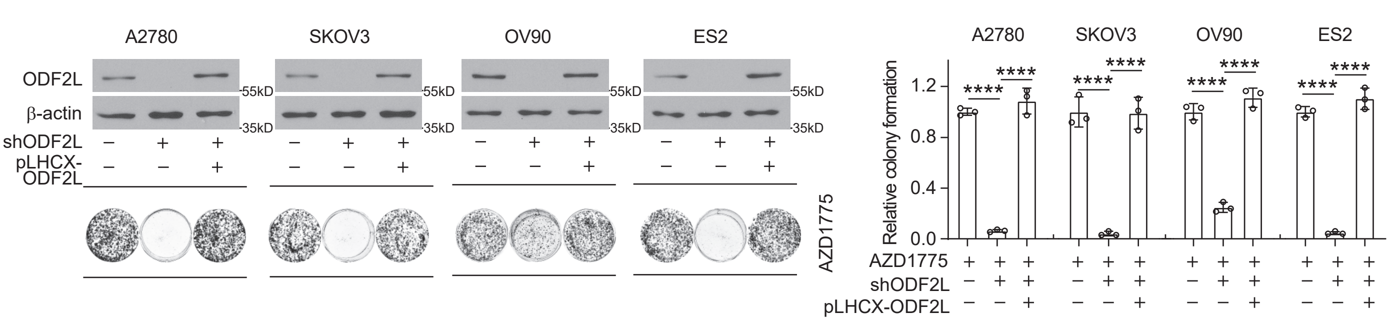

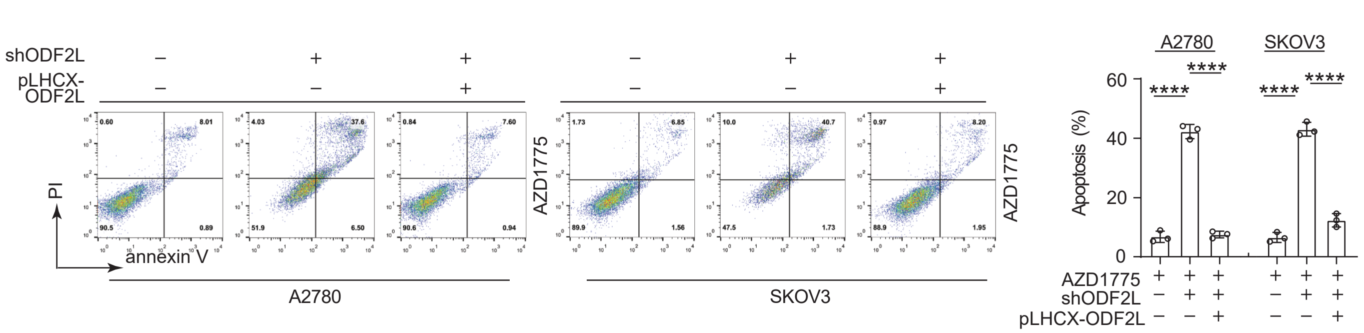

Supplemental Figure 3

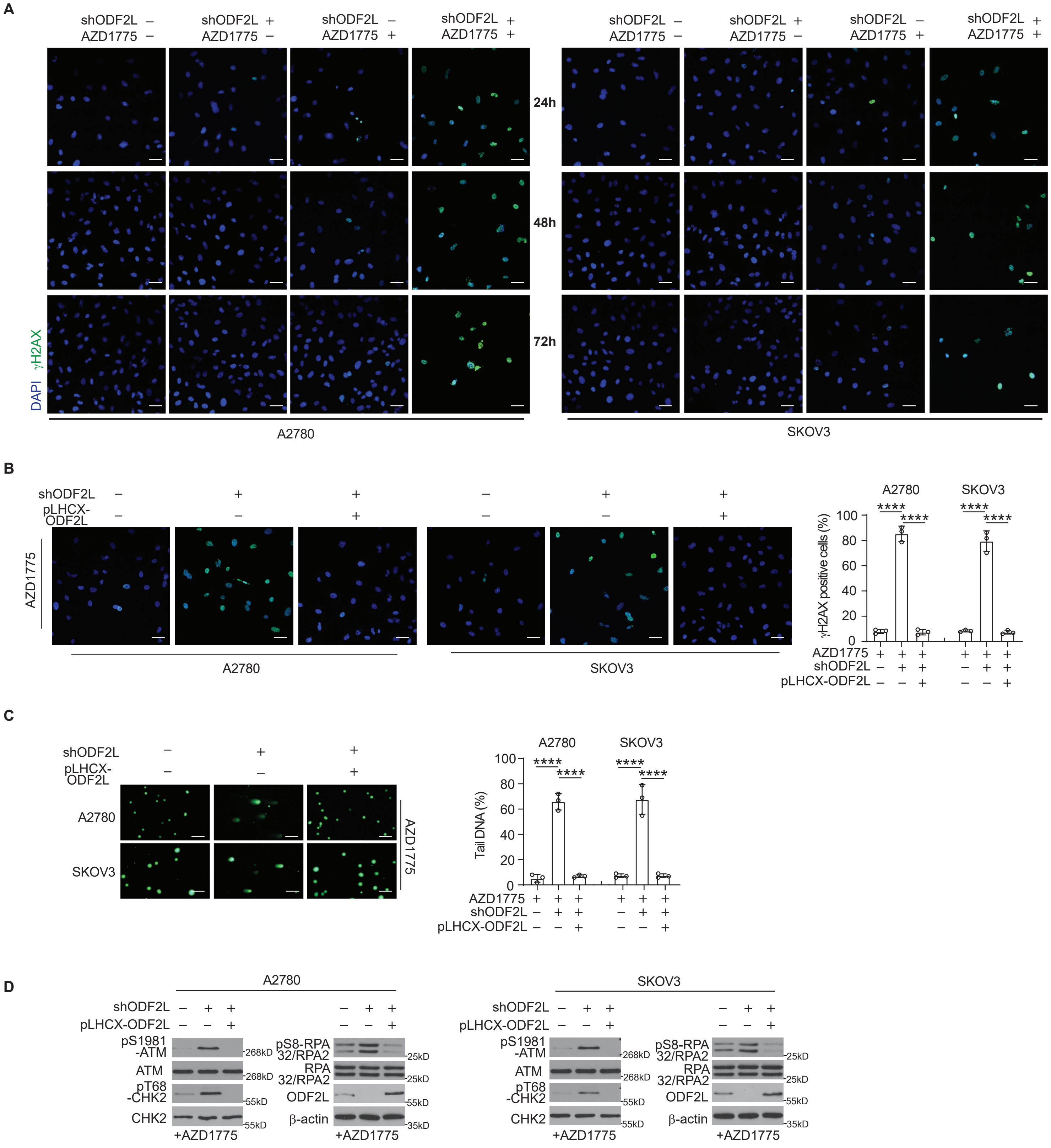

Supplemental Figure 4

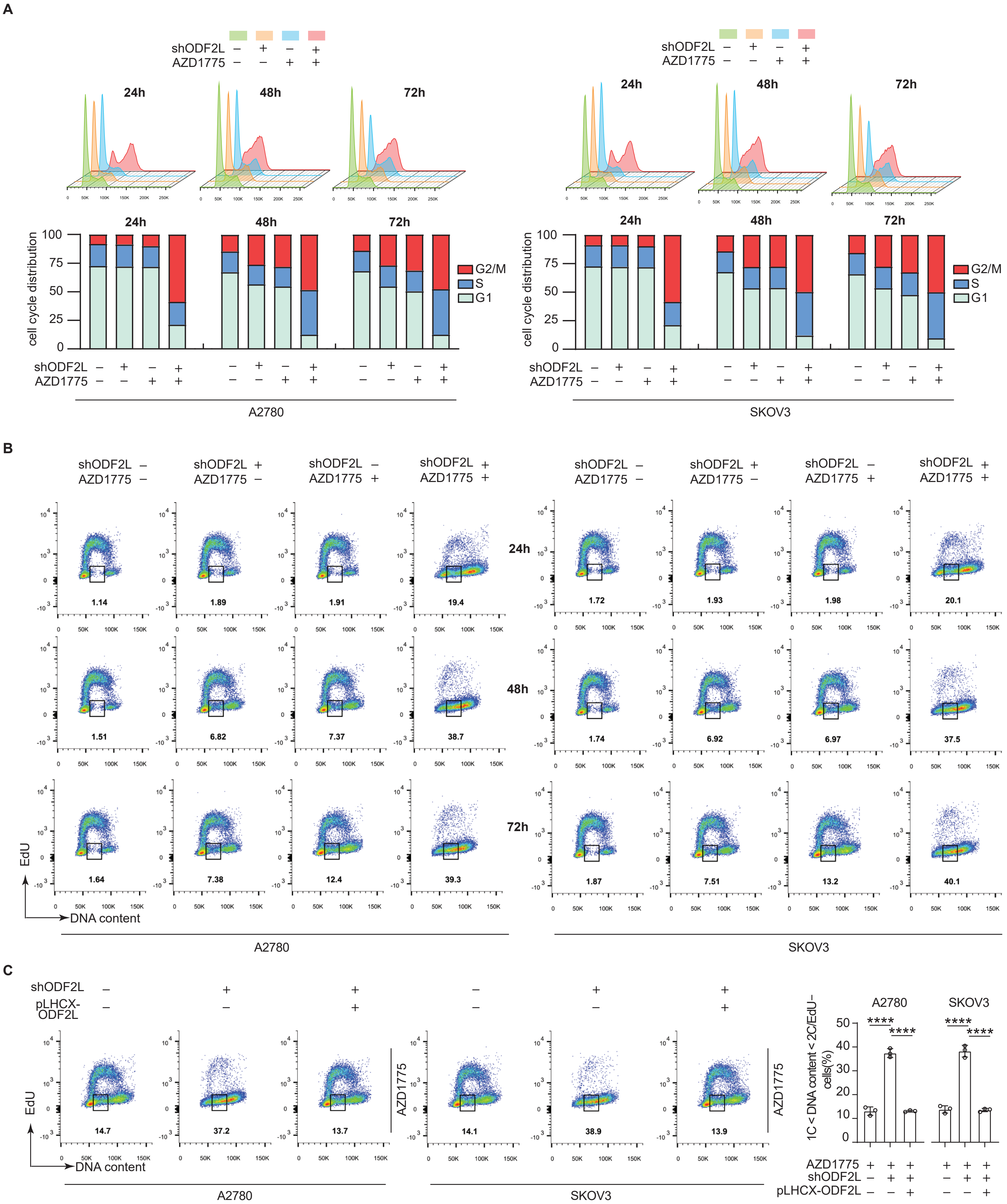

Supplemental Figure 5

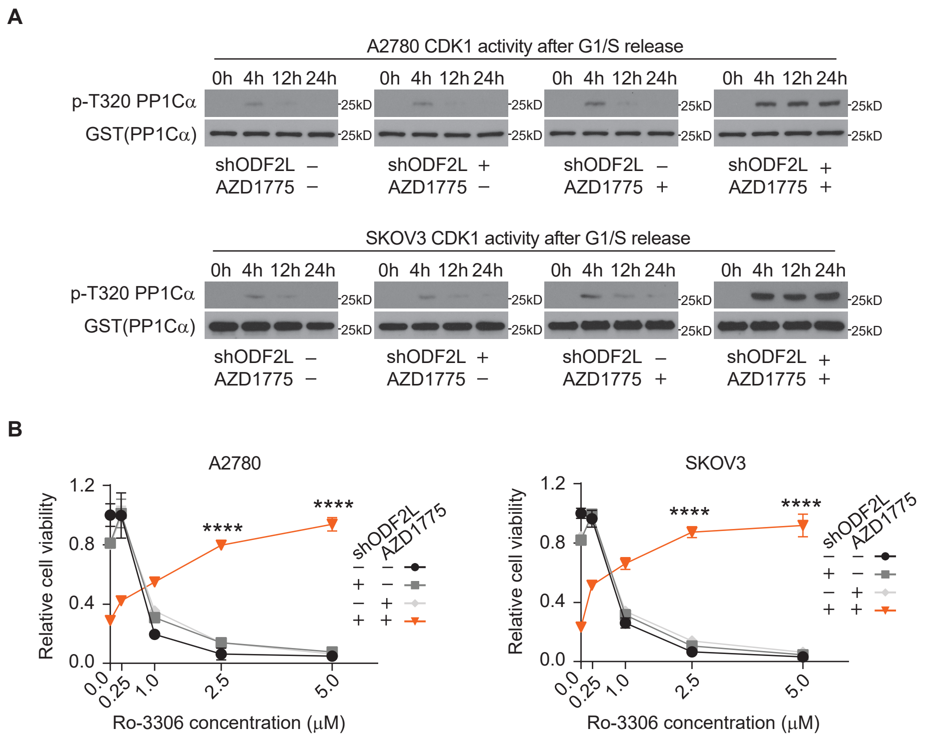

Supplemental Figure 6

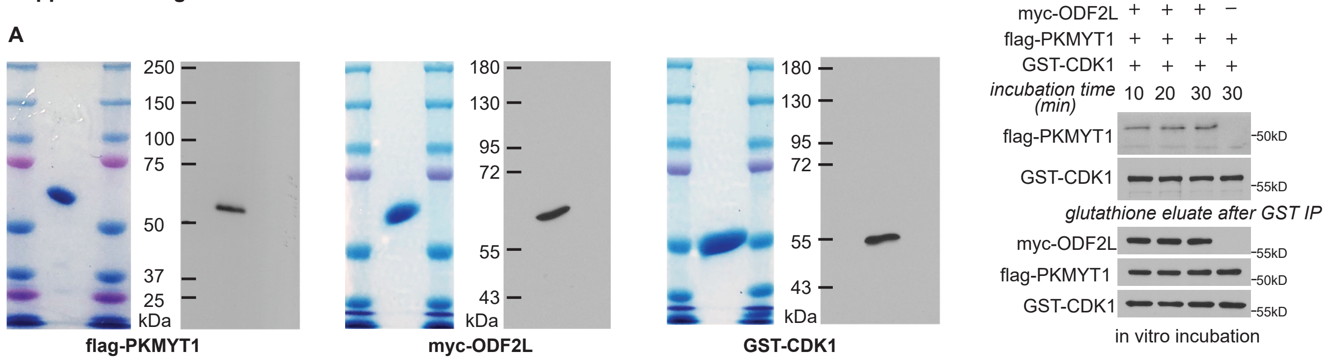

Supplemental Figure 7

A

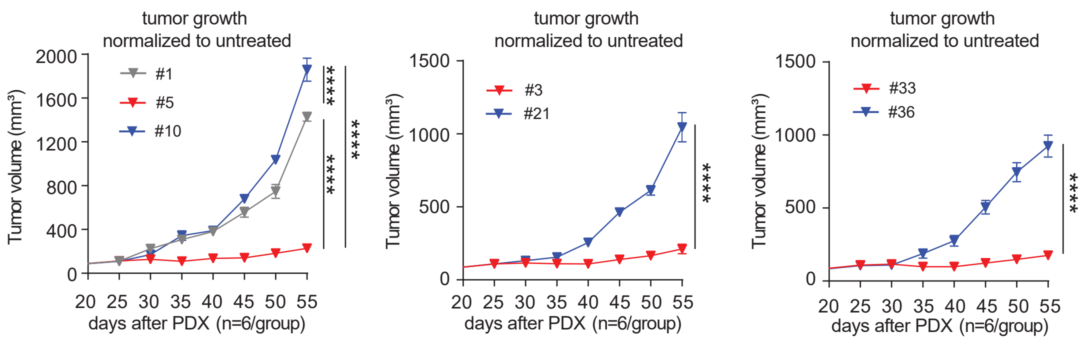

B

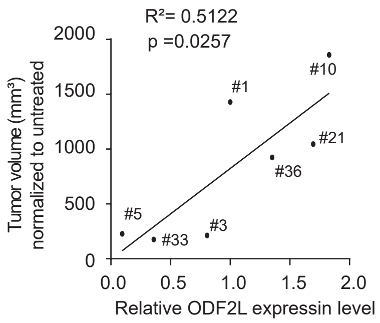

Supplemental Figure 8

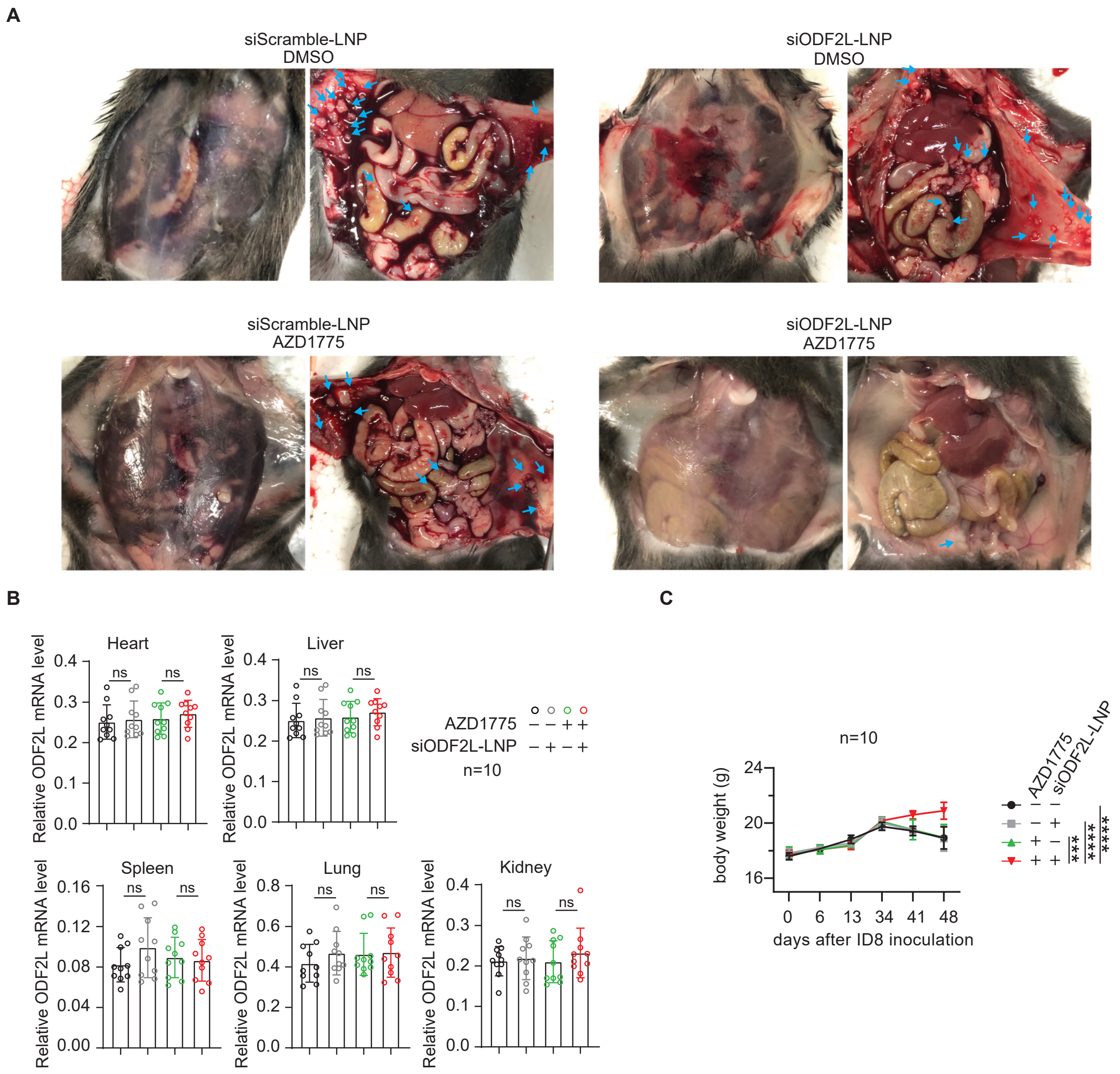

Supplement: Supplemental data [file jci-133-161544-s019.pdf]
